# An Endophytic Fungus, *Talaromyces radicus*, Isolated from *Catharanthus roseus*, Produces Vincristine and Vinblastine, Which Induce Apoptotic Cell Death

**DOI:** 10.1371/journal.pone.0144476

**Published:** 2015-12-23

**Authors:** Padmini P. C. Palem, Gini C. Kuriakose, Chelliah Jayabaskaran

**Affiliations:** Department of Biochemistry, Indian Institute of Science, Bangalore, India; The University of Wisconsin - Madison, UNITED STATES

## Abstract

Endophytic fungi isolated from *Catharanthus roseus* were screened for the production of vincristine and vinblastine. Twenty-two endophytic fungi isolated from various tissues of *C*. *roseus* were characterized taxonomically by sequence analysis of the internal transcribed spacer (ITS) region of rDNA and grouped into 10 genera: *Alternaria*, *Aspergillus*, *Chaetomium*, *Colletotrichum*, *Dothideomycetes*, *Eutypella*, *Eutypa*, *Flavodon*, *Fusarium* and *Talaromyces*. The antiproliferative activity of these fungi was assayed in HeLa cells using the MTT assay. The fungal isolates *Eutypella sp*—CrP14, obtained from stem tissues, and *Talaromyces radicus*—CrP20, obtained from leaf tissues, showed the strongest antiproliferative activity, with IC_50_ values of 13.5 μg/ml and 20 μg/ml, respectively. All 22 endophytic fungi were screened for the presence of the gene encoding tryptophan decarboxylase (TDC), the key enzyme in the terpenoid indole alkaloid biosynthetic pathway, though this gene could only be amplified from *T*. *radicus*—CrP20 (NCBI GenBank accession number KC920846). The production of vincristine and vinblastine by *T*. *radicus*—CrP20 was confirmed and optimized in nine different liquid media. Good yields of vincristine (670 μg/l) in modified M2 medium and of vinblastine (70 μg/l) in potato dextrose broth medium were obtained. The cytotoxic activity of partially purified fungal vincristine was evaluated in different human cancer cell lines, with HeLa cells showing maximum susceptibility. The apoptosis-inducing activity of vincristine derived from this fungus was established through cell cycle analysis, loss of mitochondrial membrane potential and DNA fragmentation patterns.

## Introduction

*Catharanthus roseus* (*Vinca rosea*), a short-lived perennial belonging to the Apocynaceae family, produces more than one hundred terpenoid indole alkaloids (TIAs) of medicinal value, and this plant has long been an integral part of ancient Ayurveda [1]. Vincristine (VCR; Oncovin) and vinblastine (VBL; Velbe) are the two major vinca alkaloids most widely used in chemotherapy regimens for the treatment of a variety of solid tumors as well as Hodgkin’s disease and leukemia. These anti-cancer alkaloids inhibit cell proliferation by binding to microtubules, leading to mitotic block and apoptosis [2], and they contain a bis-indole nitrogen moiety derived from tryptophan in the TIA pathway [3]. The first critical step in the biosynthesis of TIAs is a reaction catalyzed by tryptophan decarboxylase (TDC), which is encoded by a single gene in *C*. *roseus* [4]. The green glossy leaves of *C*. *roseus* are the only known source of VCR and VBL; however, the yields are low, and meeting the estimated annual worldwide demand of 3 kg requires processing a massive 300 tons of dried leaves [5]. Accordingly, the increasing demand for VCR and VBL has encouraged research into improved production methods involving cell culture, metabolic engineering [6], and semi- and total chemical synthesis [7]. The fact that these plants have low VCR and VBL contents, are difficult and expensive to process, and require a long time to propagate [8] has prompted a search for sustainable, economical and unconventional alternative sources of these compounds. The isolation of the taxol-producing endophytic fungus *Taxomyces andreanae* from *Taxus brevifolia* has piqued interest in endophytic fungi as potential sources of chemotherapeutic agents [9]; thus, plant endophytic fungi may represent a viable option for the production of TIAs [10]. Furthermore, fungal endophytes have the ability to produce bioactive compounds [11] and autonomously synthesize secondary metabolites [12] similar to those of the host plant. For instance, studies have shown that several endophytic fungi isolated from *Taxus* species can produce taxol [13, 14], and camptothecin-producing and podophyllotoxin-producing endophytic fungi have been identified from *Camptotheca accuminata* [15] and *Podophyllum peltatum* [16], respectively. In addition, reports from China claim that VBL can be produced by an unidentified endophytic fungus [17] and that VCR can be produced by *Fusarium oxysporum* [18] isolated from *C*. *roseus*. Recently, endophytic fungi grown in a medium supplemented with the precursor L-tryptophan have been reported to produce VCR and VBL [19, 20]. Although it is possible that fungi may harbor the required biosynthetic gene and that vinca alkaloids of fungal origin have apoptotic potential, experimental evidence is needed to confirm these possibilities.

The objectives of the present investigation were to isolate endophytic fungi from *C*. *roseus*, to screen the fungi for the presence of the TDC gene and the production of vinca alkaloids, to optimize the production of VCR and VBL using different growth media and to reconfirm the cytotoxic and apoptotic efficiencies of these compounds.

## Materials and Methods

### Isolation of endophytic fungi from *C*. *roseus*

Samples of *C*. *roseus* were collected from the nursery of the Indian Institute of Science, Bangalore, India. The plant material was identified by Prof. K. Sankar Rao, a plant taxonomist, at the Center for Ecological Sciences, Indian Institute of Science, Bangalore. Fresh plant material was treated with commercial bleach (20%) and Tween 20 (0.1%) for 5 min and rinsed with sterilized distilled water. Plant parts, i.e., roots, stems, leaves and flowers, were then soaked in a solution containing bavistin (30 mg), tetracycline (0.6 mg) and Tween 20 (0.1%), washed, and treated with HgCl_2_ (0.1%) for 10 min. The plant parts were cut into small segments using a sterilized sharp blade, placed in an upright position in potato dextrose agar (PDA) medium in Petri plates and incubated at 25 ± 2°C. The fungal mycelia that emerged from the cut surface of the segments after a few days were transferred onto fresh PDA medium and incubated at 25 ± 2°C for 10 days. The fungal cultures were checked for purity using the single hyphal tip method and stored as spore and mycelial stocks in 15% glycerol at -70°C.

### Identification of endophytic fungi

#### DNA extraction

Fungal cultures were grown in potato dextrose broth (PDB) at 25 ± 2°C for 5 days, and mycelia were harvested by filtration through cheesecloth. The mycelia were then blotted dry, and the mycelial mass of individual fungal isolates was pulverized in a pre-cooled mortar and pestle with liquid nitrogen. Total genomic DNA was extracted using a method described elsewhere [21], and the concentration was determined by measuring the absorbance at 260 nm using a UV-vis spectrophotometer. Approximately 300 μg of genomic DNA was obtained from 1 g of mycelium.

#### PCR amplification of ITS rDNA and sequence analysis

Genomic polymerase chain reaction (PCR) was performed using the universal ITS1 forward primer (5’-TCCGTAGGTGAACCTGCGG-3’) and ITS4 reverse primer (5’- TCCTCCGCTTATTGATATGC-3’) [22]. The reaction mixture contained genomic DNA (100 ng), ITS primers (100 μM), dNTPs (150 μM) and Taq DNA polymerase (2.5 U) in a total volume of 50 μl. PCR was carried out using a PCR thermocycler (Minicycler TM, Germany) under the following conditions: 35 cycles of denaturation at 96°C for 45 s, annealing at 50°C for 45 s, and extension at 72°C for 45 s, followed by a final extension at 72°C for 10 min. The amplified products were separated by 0.8% agarose gel electrophoresis and purified using a PCR clean-up kit. The purified PCR products were sequenced by Xcelris Labs, Bangalore, India, using the same primers that were used for PCR. Sequence data were aligned and compared with sequences deposited in the GenBank database using the NCBI BLASTN program [23]. The fungi were assigned to a genus based on a 99–100% similarity index.

### Preparation of endophytic fungal extracts and assessment of *in vitro* antiproliferative activity by the MTT assay

Endophytic fungal cultures were initiated by transferring three 5-mm agar plugs of each fungus (10-day-old cultures grown on PDA medium) to 300 ml of PDB medium and incubated at 25 ± 2°C under static conditions. After 21 days, the mycelia and filtrate were collected and separately extracted twice with an equal volume of ethyl acetate. The mycelia were frozen in liquid nitrogen, crushed using a mortar and pestle and extracted with five volumes (w/v) of ethyl acetate. The organic phases were separated and evaporated to dryness under reduced pressure using a rotary vacuum evaporator at 40°C. The crude extract was dissolved in methanol and filtered through 0.25 μm filters.

The antiproliferative activities of the mycelial extracts and filtrate were determined in 96-well plates using MTT, as described elsewhere [24]. HeLa cells at a density of 1 x 10^4^ cells per well were seeded in 100 μl of Dulbecco’s modified Eagle’s medium (DMEM) with 10% fetal bovine serum (FBS) in 96-well plates and grown for 24 h at 37°C in a 5% CO_2_ incubator. The cells were then treated with fungal extracts at various concentrations, ranging from 5 to 100 μg/ml. After 24 h, MTT solution (10 μl of a 5 mg/ml stock) in PBS was added to each well and incubated for 2 h; the supernatant was then removed, and DMSO (100 μl) was added to each well to dissolve the formazan crystals. The absorbance was measured at 570 nm using a microplate reader.

### Genomic PCR-based screening for TIA-producing fungi

All 22 endophytic fungi obtained were screened for the presence of the TDC gene as a molecular marker for TIA-producing fungi. For this purpose, TDC gene-specific primers (F-5’-ACCTACGACCGTCGAACGC-3’ and R-5’-AAACTCGGGACATATACAGG-3’) were used [25], resulting in a 232-bp amplicon. PCR amplification was carried out in a typical 50-μl reaction mixture containing genomic DNA (100 ng), the TDC primer set (100 μM each), dNTPs (150 μM) and Taq DNA polymerase (2.5 U) in Taq buffer (1X) using a PCR thermocycler (Minicycler TM, Germany) under the following conditions: initial denaturation at 94°C for 3 min; 30 cycles of denaturation at 94°C for 1 min, annealing at 57.5°C for 1 min, and extension at 72°C for 1 min; and a final extension at 72°C for 5 min. The amplified DNA fragment was analyzed by agarose gel electrophoresis.

### Morphological observation of the TDC gene-positive fungal isolate *T*. *radicus*—CrP20

Strain CrP20 was grown on the surface of PDA for 10 days at 25°C for identification based on fungal colony morphology and characterization of the conidia. The lactophenol cotton blue staining method was used to stain the fungal culture growing under a glass coverslip. The culture was visualized using light microscopy (Carl Zeiss, USA) and then photographed.

### Isolation and identification of VCR and VBL in extracts of *T*. *radicus*—CrP20

It has been suggested that specific methods involving acid and base extraction are required for the recovery of alkaloids [26–28]. Hence, the following protocol was standardized to recover the maximum amount of each compound. The fungal isolate was grown in PDB at 25 ± 2°C. After 21 days, the entire culture (medium plus mycelium) was thoroughly blended, and the pH was adjusted to 2.0 with H_2_SO_4_ (1 N); the sample was then extracted twice with an equal volume of dichloromethane, retaining the aqueous extract each time. After increasing the pH of the aqueous phase to 9.5 with NaOH (1 N), alkaloids were extracted twice with an equal volume of dichloromethane. The combined organic phases were then evaporated under reduced pressure at 40°C, and the residue was dissolved in 1 ml of HPLC-grade methanol.

The methanolic extract was subjected to thin-layer chromatography (TLC) on Merck 0.25 mm silica gel plates in a solvent system consisting of chloroform:methanol (7:3) along with VCR and VBL standard solutions (Sigma Aldrich, USA). The fungal VCR and VBL as well as the reference standards were visualized on the TLC plates using alkaloid-specific Dragendorff’s reagent.

The molecular masses of fungal VCR and VBL were determined by subjecting the crude fungal extracts to LC-ESI-MS (M/S applied Thermo LCQ Deca XP Max ESI-MS analyzer) along with VCR and VBL standards as the reference compounds. The mobile phase used was acetonitrile:acetic acid (1%) in a 20:80 ratio with a flow rate of 0.5 ml/min, an injection volume of 10 μl and an IS voltage of 4,500 V in positive mode. A spectrum with a range of m/z 200–1000 was applied, and the molecular ions of fungal VCR and VBL in the samples were analyzed along with those of the VCR and VBL standards, which served as the reference compounds.

### Quantification of *T*. *radicus*—CrP20 VCR and VBL in different media

The production of VCR and VBL by the TDC gene-positive fungal isolate was evaluated using nine different media. Table 1 shows the composition of all media used for optimization. Each recipe shows the components for one liter of medium, which was autoclaved for 20 min at 121°C and cooled overnight before inoculation. All experiments were conducted in 300 ml of each medium and extracted as described above. Crude fungal extracts (20 mg each) were dissolved in 1 ml of HPLC-grade methanol and injected into an Agilent C18 HPLC column (length of 4.6 x 150 mm, pore size of 3 μm and particle size of 120 A). The instrument used was a Surveyor PDA detector (Thermo Finnigan) with an applied wavelength of 252 nm. After injecting 20 μl of each sample, a gradient elution of 10–100% was performed using formic acid (0.1%) in 5 mM ammonium acetate (solution A) and methanol (solution B) at a flow rate of 0.8 ml/min. Different concentrations of VCR and VBL standard solutions were also analyzed to construct a standard curve. The peak areas of different concentrations of the standard solutions were used to quantify fungal VCR and VBL per liter of total culture.

**Table 1:** Different media used for the optimization of fungal VCR and VBL production.

Liquid media	pH	Composition (g.L^-1^)
Potato dextrose broth (PDB)	6.0	Potato infusion 4.0; dextrose 20
M1D	5.6	Sucrose 12; ammonium tartarate 5; yeast extract 0.5; calcium nitrate 0.28; potassium nitrate 0.08; potassium chloride 0.06; magnesium sulfate 0.36; sodium phosphate 0.02; boric acid 0.014; manganese sulfate 0.05; zinc sulfate 0.025; potassium iodide 0.007; ferric chloride 0.002
Flask basal medium (FBM)	7.0	Glucose 80; ammonium nitrate 5; magnesium sulfate 0.5; potassium phosphate 0.5; zinc sulfate 0.001; copper nitrate 0.001; ferric chloride 0.002; sodium acetate 1; vitamin B1 0.05
Modified flask basal medium (MFBM)	7.0	Glucose 80; ammonium nitrate 8; magnesium sulfate 0.7; potassium phosphate 0.6; zinc sulfate 0. sulfate; copper nitrate 0.001; ferric chloride 0.002; sodium acetate 2; vitamin B1 0.05
M2	6.0	Glucose 18; peptone 0.45; yeast extract 0.9; ammonium sulfate 2.7; potassium dihydrogen phosphate 1.8; magnesium sulfate 0.45
Modified M2 (MM2)	6.0	Glucose 18; peptone 9; yeast extract 9; ammonium sulfate 2.7; potassium di-hydrogen phosphate 1.8; magnesium sulfate 0.45
M3	6.0	Sucrose 27; sodium nitrate 2.7; di-potassium hydrogen phosphate 0.9; potassium chloride 0.45, magnesium sulfate 0.45, ferrous sulfate 0.009,
Modified M3 (MM3)	6.0	Sucrose 27; peptone 9; yeast extract 9; sodium nitrate 2.7; di-potassium hydrogen phosphate 0.9; potassium chloride 0.45, magnesium sulfate 0.45, ferrous sulfate 0.009
S7	7.0	Glucose 1; fructose 3; sucrose 6; yeast extract 0.5; zinc sulfate 0.0025; ferric chloride 0.0027; manganese sulfate 1; calcium nitrate 0.0006; sodium benzoate 0.05; sodium acetate 0.1

### Apoptotic activity of fungal VCR

#### Preparation of ‘fungal VCR’ for assessment of apoptotic activity

The fungal extract prepared from the TDC gene-positive isolate was first separated by TLC. The spot corresponding to VCR was scraped and eluted with methanol. The resulting partially purified material, referred to as ‘fungal VCR’, was used in further studies.

#### Human cancer cell lines and culture conditions

HeLa (human cervical carcinoma), MCF7 (human breast adenocarcinoma), A549 (human lung cancer), U251 (human neuronal glioblastoma) and A431 (melanoma) cell lines were procured from the National Centre for Cell Sciences (NCCS) in Pune, India. The cell lines were cultured in DMEM supplemented with FBS (10%) in a 5% CO_2_ incubator at 37°C. Penicillin (100 μg/ml) and streptomycin (100 μg/ml) were added to prevent bacterial growth.

#### Cytotoxic effects of ‘fungal VCR’ on human cancer cell lines

The cytotoxicity of the ‘fungal VCR’ toward human cancer cell lines was determined by the MTT assay. To measure inhibition of cell proliferation after treatment with fungal VCR, the standard MTT assay was performed as described above.

#### Effect of ‘fungal VCR’ on cell cycle distribution in HeLa cells

The effect of the ‘fungal VCR’ on cell cycle distribution was analyzed using flow cytometry [29]. HeLa cells were seeded at a density of 2.5 x 10^5^ cells per well in 24-well plates containing 0.5 ml of culture medium and incubated for 24 h. The cells were treated with different concentrations of ‘fungal VCR’ (5, 10 and 25 μg/ml) in DMEM supplemented with FBS (10%). After a 24-h incubation, the cells were harvested, washed twice with ice-cold PBS, and fixed in ice-cold ethanol (70% v/v) for 30 min. The cells were then stained in PBS containing propidium iodide (20 μg/ml), RNaseA (100 μg/ml) and Triton X-100 (1%). After 60 min at 37°C, the cells were analyzed with FACScan using a FACSCalibur flow cytometer (Becton Dickinson, USA). Data were analyzed using CellQuest Pro software. Apoptotic cell populations were quantified based on the sub-G_1_ peak.

#### Analysis of phosphatidyl serine externalization by HeLa cells treated with ‘fungal VCR’ using annexin V-FITC and PI dual staining

The apoptotic cell death induced by ‘fungal VCR’ was measured by flow cytometry using annexin V-FITC/ propidium iodide (PI) staining according to a procedure described elsewhere [30]. Approximately 2.5 x 10^5^ cells per well were seeded in 24-well plates and treated with different concentrations (5, 10 and 25 μg/ml) of ‘fungal VCR’ for 24 h in DMEM FBS medium in a 5% CO_2_ incubator. The cells were then stained with annexin V-FITC and PI and analyzed using a FACSCalibur flow cytometer (Becton Dickinson, USA). The data obtained at an excitation (ƛ_ex_)/emission (ƛ_em_) of 488/520 nm for annexin V-FITC and 540/630 nm for PI were analyzed using CellQuest Pro software.

#### Induction of HeLa cell mitochondrial membrane depolarization by ‘fungal VCR’

Changes in the mitochondrial membrane potential after treatment with ‘fungal VCR’ were determined by the JC-1 staining assay [31]. Briefly, 2.5 x 10^5^ HeLa cells per well were incubated in 24-well plates containing DMEM FBS medium. After 2 h, the cells were treated with different concentrations (5, 10 and 25 μg/ml) of ‘fungal VCR’ for 24 h, stained with 2.5 μg/ml of JC-1 dye and incubated for 15 min at 37°C in a CO_2_ incubator. In parallel, cells were also treated with valinomycin (25 μM) as a positive control. The cells were washed with ice-cold PBS containing FBS (2% v/v), resuspended in the same buffer, and immediately analyzed by flow cytometry for red and green fluorescence using a FACSCalibur flow cytometer (Becton Dickinson, USA). Data were analyzed using CellQuest Pro software.

#### DNA fragmentation assay in HeLa cells

A DNA fragmentation assay was performed as described previously [32]. Briefly, 4 x 10^6^/ml of HeLa cells were cultured in the presence of 10 and 25 μg/ml fungal VCR for 24 h. The treated cells were centrifuged, lysed and incubated with RNase A (3 μg/ml) for 30 min at 37°C followed by incubation with proteinase K (3 μg/ml) at 37°C for 30 min. The DNA samples were electrophoresed through a horizontal agarose gel (0.8%) and visualized by staining the gel with ethidium bromide (5 μg/ml) and examining it under UV light.

## Results

### Isolation and identification of endophytic fungi from *C*. *roseus*

**Fig 1:**
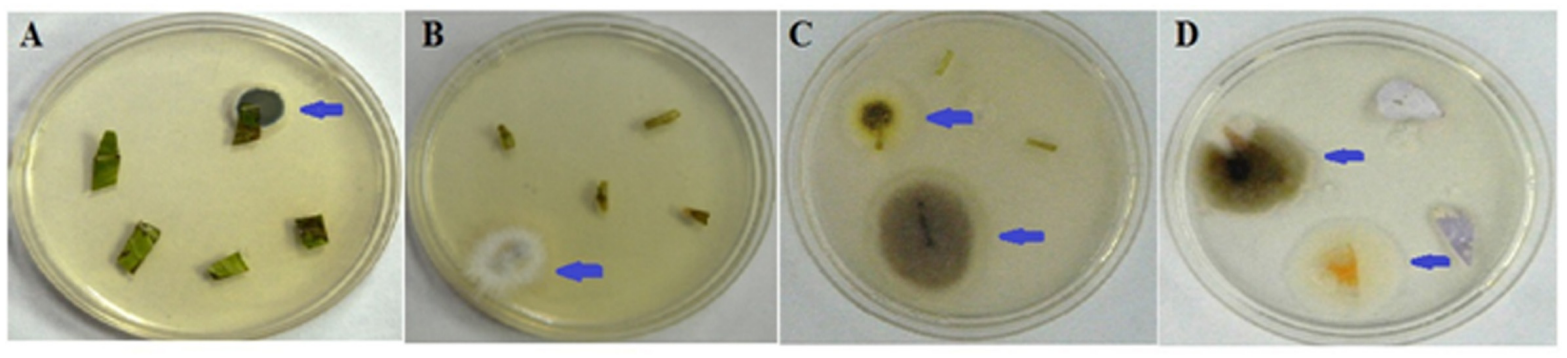
Isolation of endophytic fungi from *C*. *roseus* plant. Arrowheads indicate the emergence of endophytic fungi from the plant cuttings. A-leaf, B-stem, C-pedicel and D-flower petal.

**Fig 2:**
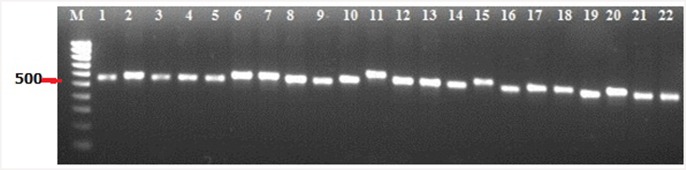
PCR products of approximately 550 to 600 bp of ITS region in endophytic fungi of *C*. *roseus*. Lane M: 100-bp DNA marker. Lanes 1 to 22: PCR products of CrP1 to CrP22.

The purpose of the present study was to identify *C*. *roseus*-associated endophytic fungi that produce anticarcinogenic compounds, with the goal of metabolically engineering these isolates to improve the yields of these compounds. A total of 22 endophytic fungi were isolated from four tissues (stem, leaf, flower petal and pedicel) of *C*. *roseus* (Fig 1). DNA from the isolates was amplified using the primers ITS1 and ITS4 to yield the ITS rDNA sequence of regions 1 and 2; this sequence also includes the 5.8S rDNA gene. A PCR product of approximately 550 to 600 bp was obtained for all isolates (Fig 2), and these products were sequenced and compared with known ITS sequences in NCBI GenBank. A total of ten unique genotypes were found: *Alternaria*, *Aspergillus*, *Chaetomium*, *Colletotrichum*, *Dothideomycetes*, *Eutypella*, *Eutypa*, *Flavodon*, *Fusarium* and *Talaromyces* (Table 2).

**Table 2:** Details of the endophytic fungi isolated from *C*. *roseus* and the antiproliferative effects (IC_50_ values) of their crude extracts against human HeLa cells.

Fungal isolate code^a^	Identified fungi^b^	Plant organ^c^	GenBank accession numbers	Cytotoxic activity IC_50_ ^d^(μg/mL) of filtrate extracts	Cytotoxic activity IC_50_ ^d^(μg/mL) of mycelia extracts
CrP1	*Alternaria alternata*	F	KC503496	50	>100
CrP2	*Colletotrichum kahawae*	F	KC503497	>100	>100
CrP3	*Colletotrichum gloeosporioides*	F	KC503498	68.5	>100
CrP4	*Colletotrichum gloeosporioides*	F	KC920830	25	>100
CrP5	*Alternaria tenuissima*	P	KC920831	>100	100
CrP6	*Aspergillus niger*	P	KC920832	>100	>100
CrP7	*Aspergillus niger*	P	KC920833	25.5	>100
CrP8	*Colletotrichum gloeosporioides*	P	KC920834	70	>100
CrP9	*Alternaria tenuissima*	P	KC920835	47.5	92.5
CrP10	*Colletotrichum kahawae*	P	KC920836	>100	100
CrP11	*Flavodon flavus*	S	KC920837	>100	>100
CrP12	*Aspergillus niger*	S	KC920838	23	>100
CrP13	*Aspergillus niger*	S	KC920839	>100	100
CrP14	*Eutypella Species*	S	KC920840	13.5	100
CrP15	*Dothideomycetes Species*	S	KC920841	>100	>100
CrP16	*Eutypa species*	S	KC920842	>100	>100
CrP17	*Talaromyces radicus*	S	KC920843	52	46
CrP18	*Talaromyces radicus*	L	KC920844	>100	>100
CrP19	*Chaetomium globosum*	L	KC920845	77.5	>100
CrP20	*Talaromyces radicus*	L	KC920846	20	13
CrP21	*Fusarium solani*	S	KC920847	39.5	>100
CrP22	*Alternaria species*	F	KC920848	49.5	>100

^a^ Endophytic fungi with code CrP were isolated from *C*. *roseus*.

^b^ Identification based on partial ITS sequence analysis

^c^ S, L, F, and P represent stem, leaf, flower petal and pedicel tissues, respectively.

^d^ IC_50_ values were calculated using Graph Pad Prism software

### Antiproliferative activity of endophytic fungal culture extracts

**Fig 3:**
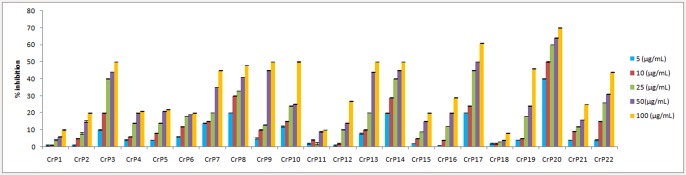
Effects of the ethyl acetate extracts of mycelium on the antiproliferative activity of HeLa cells.

**Fig 4:**
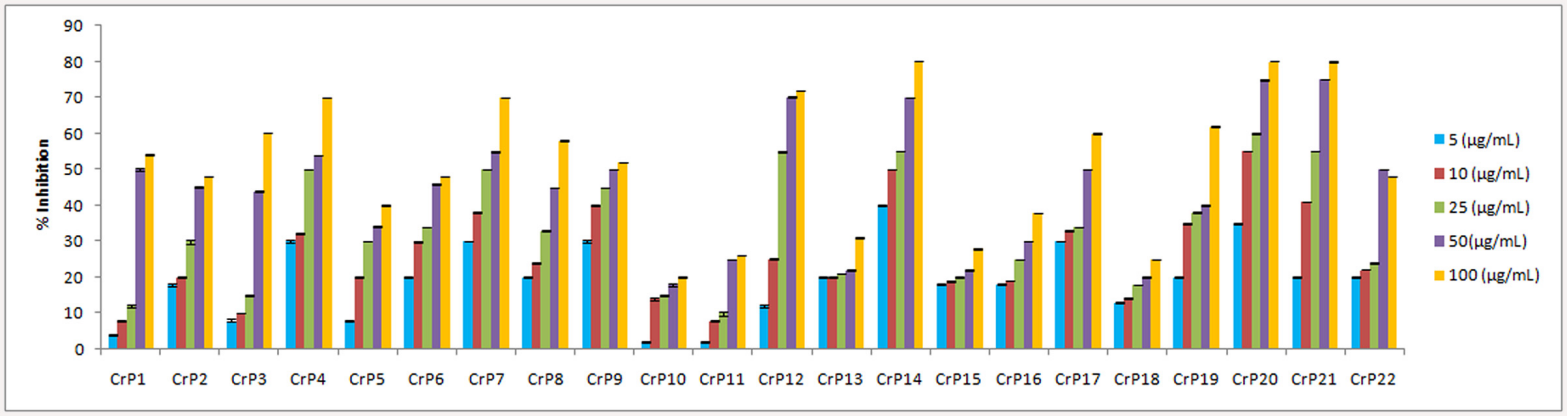
Effects of the ethyl acetate extracts of filtrate on the antiproliferative activity of HeLa cells.

Each fungal strain was cultivated in PDB, and after 21 days, fungal mycelia and the culture filtrate were extracted separately with ethyl acetate. The crude extracts were then assessed for antiproliferative activity against the HeLa human cervical cancer cell line. Five different concentrations (5–100 μg/ml) were tested for each sample to assess the dose-dependent response. All fungal samples displayed antiproliferative activity, though isolates belonging to the same genus displayed differences in inhibition (Figs 3 and 4). In the case of the mycelial extracts, two isolates of *T*. *radicus*, CrP20 and CrP17, exhibited strong antiproliferative activity, with IC_50_ values of 13.5 μg/ml and 46 μg/ml, respectively. In addition, the filtrate extracts of *Eutypella* species (CrP14), *T*. *radicus* (CrP20), *Aspergillus niger* (CrP12) and *Colletotrichum gloeosporioides* (CrP4) showed strong anti-proliferative activity, with IC_50_ values of 13.5 μg/ml, 20 μg/ml, 23 μg/ml and 25 μg/ml, respectively (Table 2).

### Genomic PCR-based screening for TIA-producing fungi

**Fig 5:**
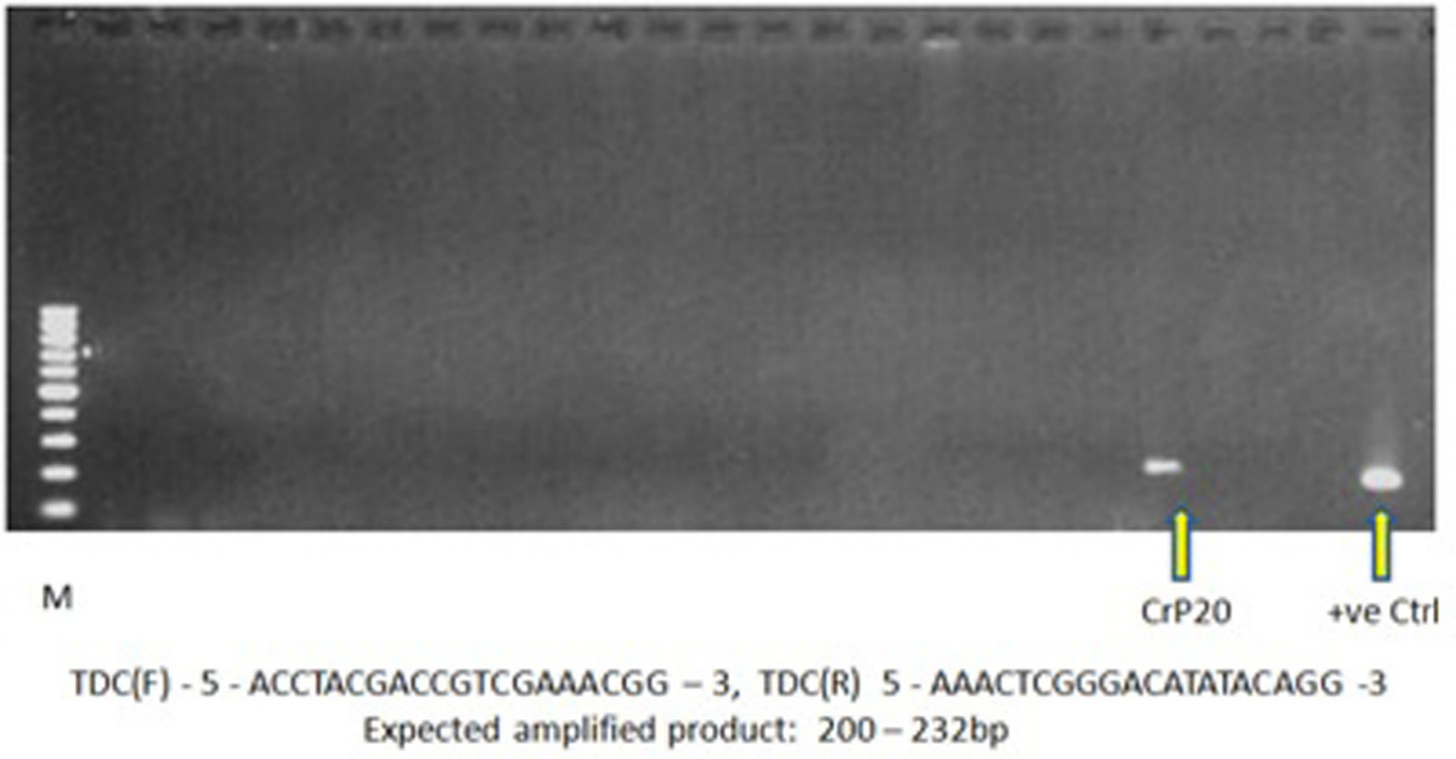
Genomic PCR analyses to determine the presence of the TDC gene. Lane M: 100-bp ladder; Lanes 1–22: the PCR amplification products of the TDC gene in 22 endophytic fungal isolates. Arrowheads show the amplified products in *T*. *radicus*—CrP20 and *C*. *roseus* (which served as the positive control).

We screened all 22 isolates by PCR amplification using TDC gene-specific primers and fungal genomic DNA to determine the presence of the TDC gene. TDC can serve as a molecular marker for identifying TIA-producing fungi. *T*. *radicus*—CrP20 was the only isolate that displayed the 232-bp amplified product of the TDC gene (Fig 5). The amplification pattern of the *T*. *radicus*—CrP20 TDC gene was comparable to that of the known standard—a *C*. *roseus* TDC gene fragment, which served as the positive control. Therefore, the *T*. *radicus*—CrP20 strain harbors the TDC gene in its genome and can be considered a potential candidate organism for vinca alkaloid production.

### Morphological assessment of the TDC gene-positive fungal isolate *T*. *radicus*—CrP20

**Fig 6:**
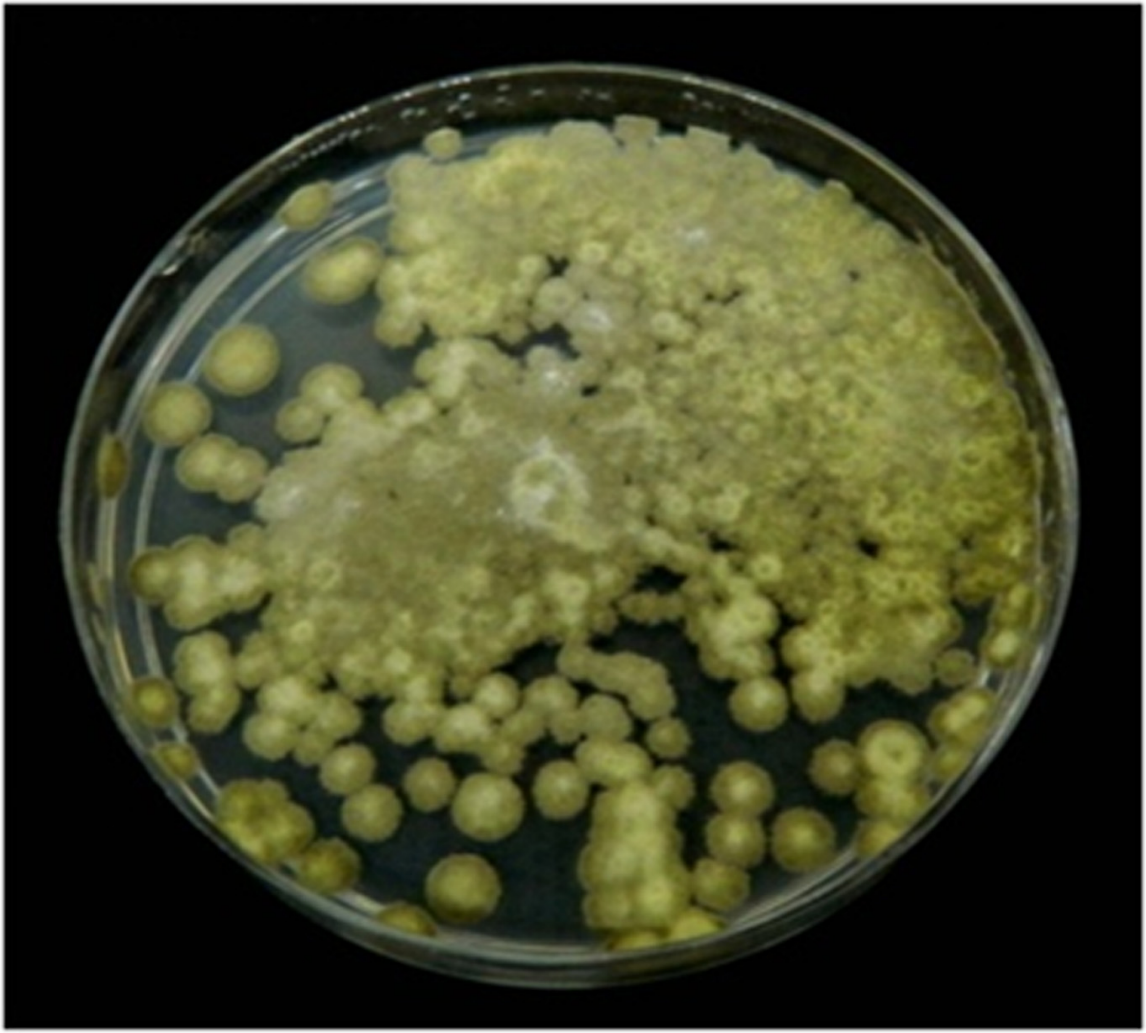
Macromorphology of Ten-day-old TIA-producing fungus *T*. *radicus*—CrP20 on PDA medium, showing mycelial growth and green-colored spores.

**Fig 7:**
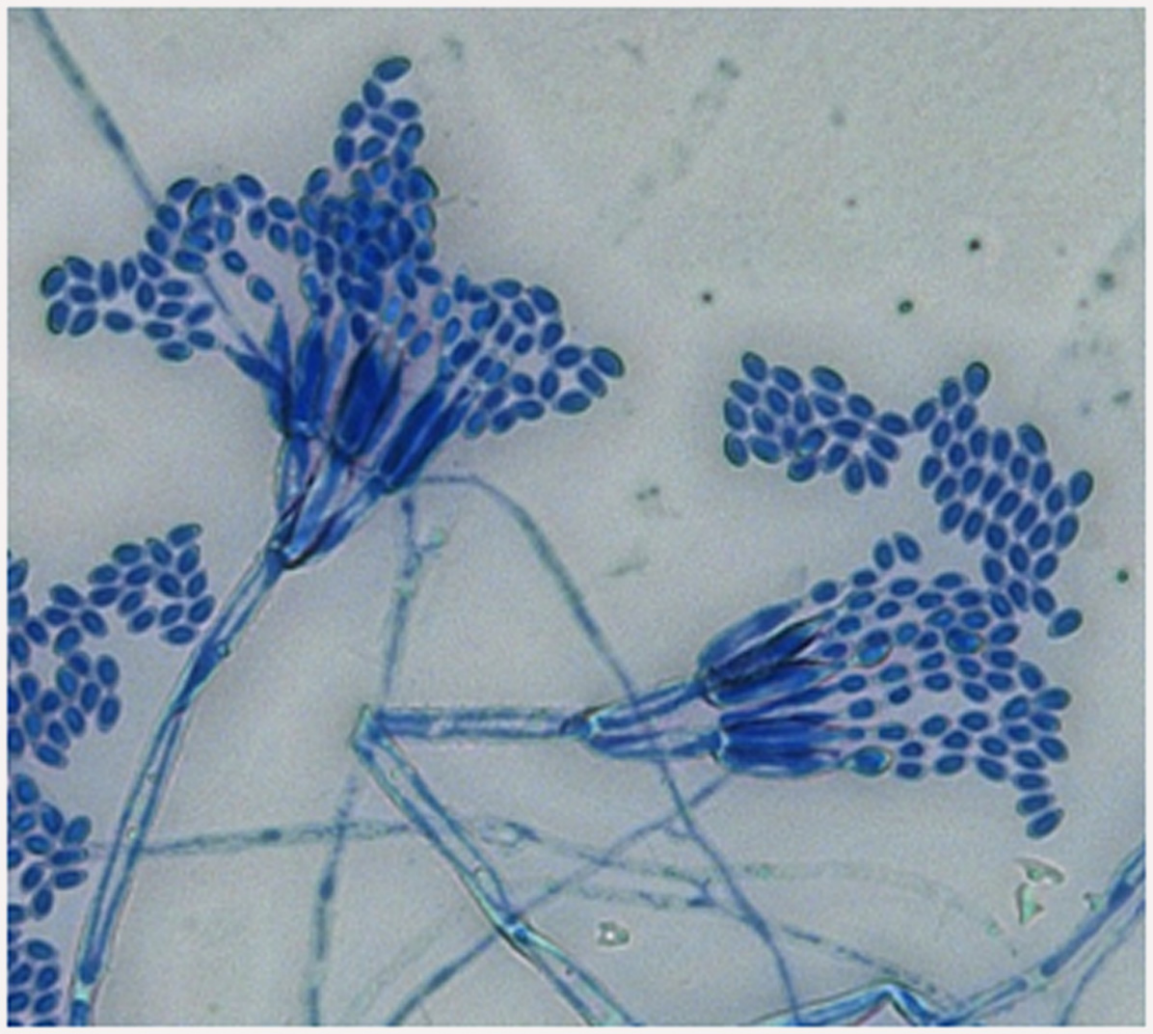
Micromorphology of *T*. *radicus*—CrP20, showing phialides with chains of conidia under light microscope (40X magnification).

After 7 days of growth on PDA medium, the isolate *T*. *radicus*—CrP20 exhibited green colonies consisting of a dense felt of conidiophores (Fig 6). Macroscopically, the colonies were green with a gray tinge, powdery, flat, dense and compact, with well-defined margins. Microscopically, the mycelia of *T*. *radicus*—CrP20 were branched, septate and hyaline. The conidial apparatus was brush shaped, consisting of ampulliform phialides. Chains of single-celled conidia with a brush-like appearance were produced in basipetal succession from branched metulae (Fig 7). The microscopic mounts were prepared in lactophenol cotton blue solution, and the fungal material was examined and photographed using a Carl Zeiss Axioskop light microscope. All the above-mentioned features of the isolate agree with the known features of genus *Talaromyces* [33].

### Isolation and identification of VCR and VBL in extracts of *T*. *radicus*—CrP20

**Fig 8:**
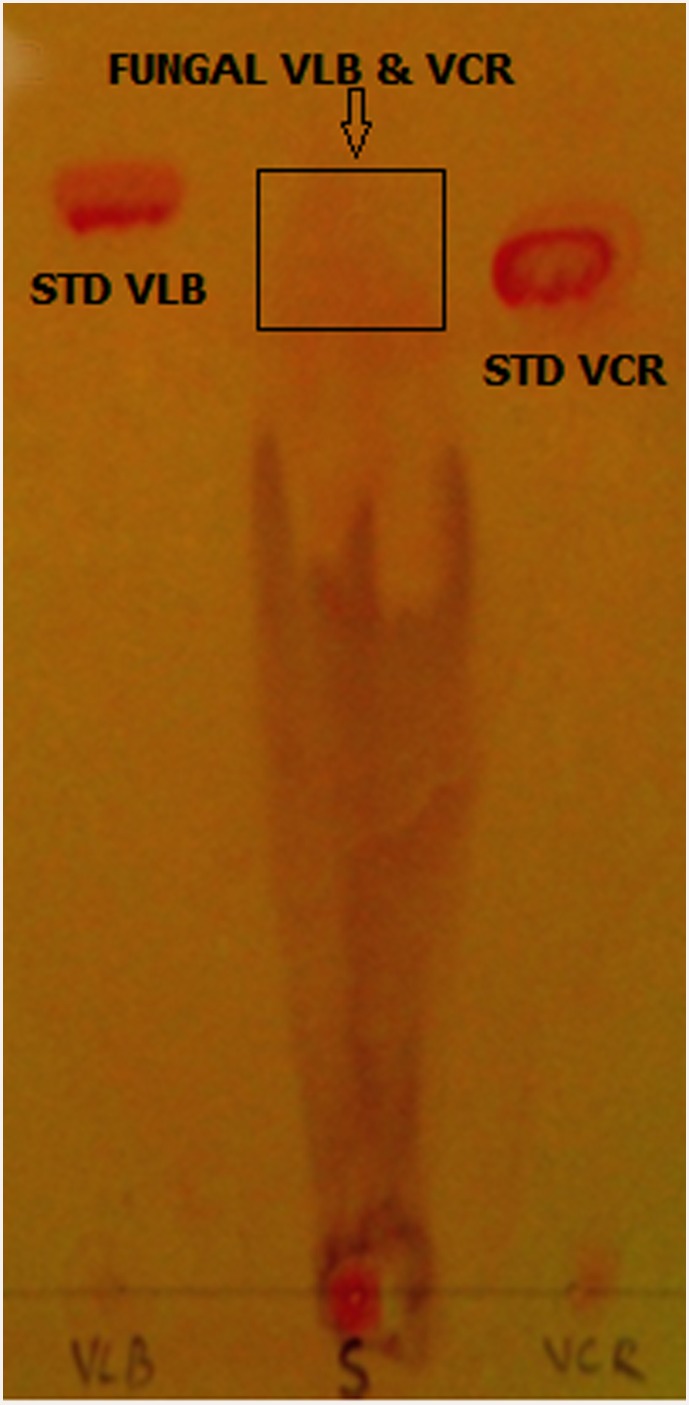
Thin-layer chromatography analysis of *T*. *radicus*—CrP20 crude extract on a silica gel aluminum sheet.

**Fig 9:**
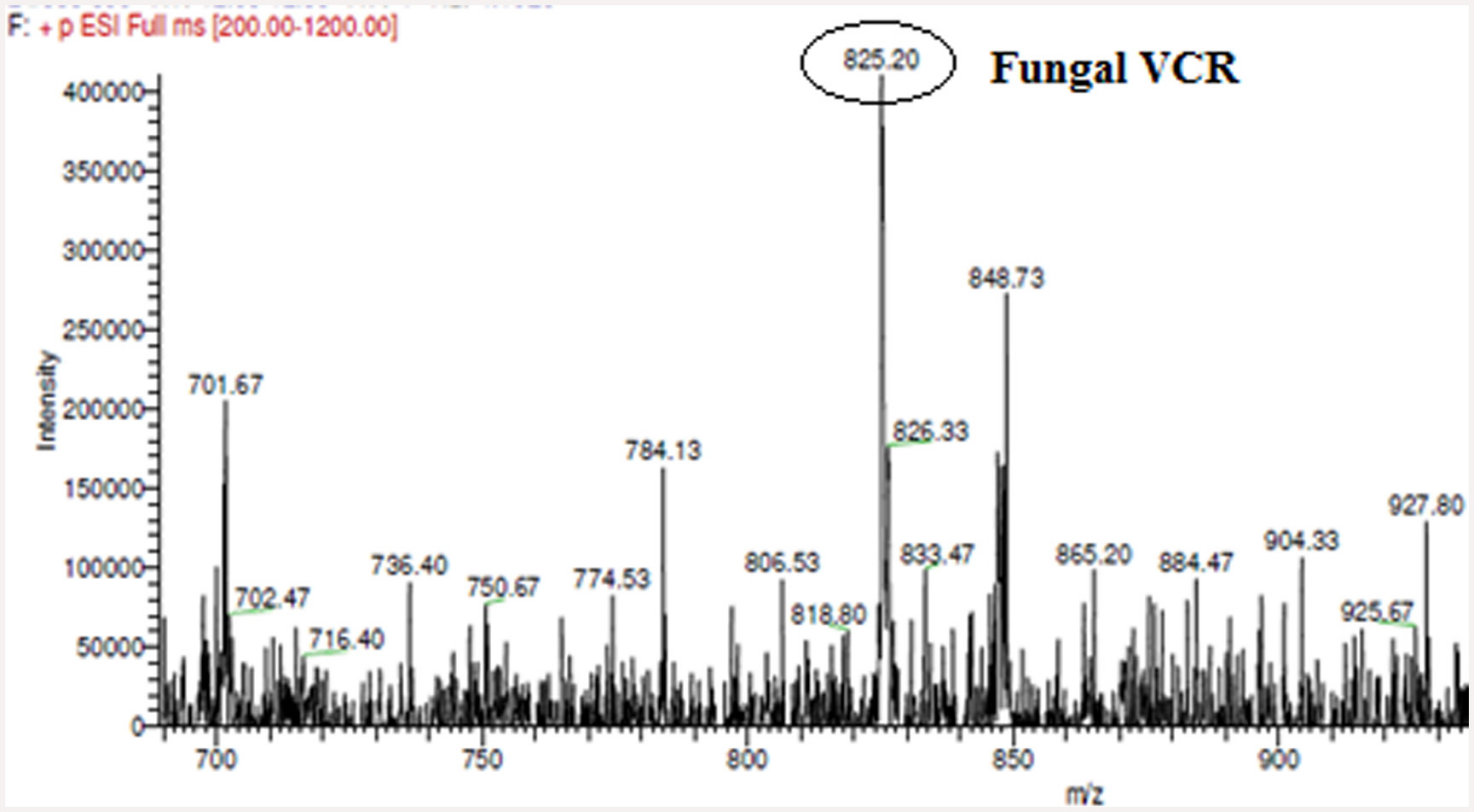
LC-ESI-MS analysis. of fungal VCR. The mass spectrum of the fungal extract showed a (M+H^+^) peak at a molecular mass of 825.46, which was identical to that observed in the mass spectrum of the VCR standard.

**Fig 10:**
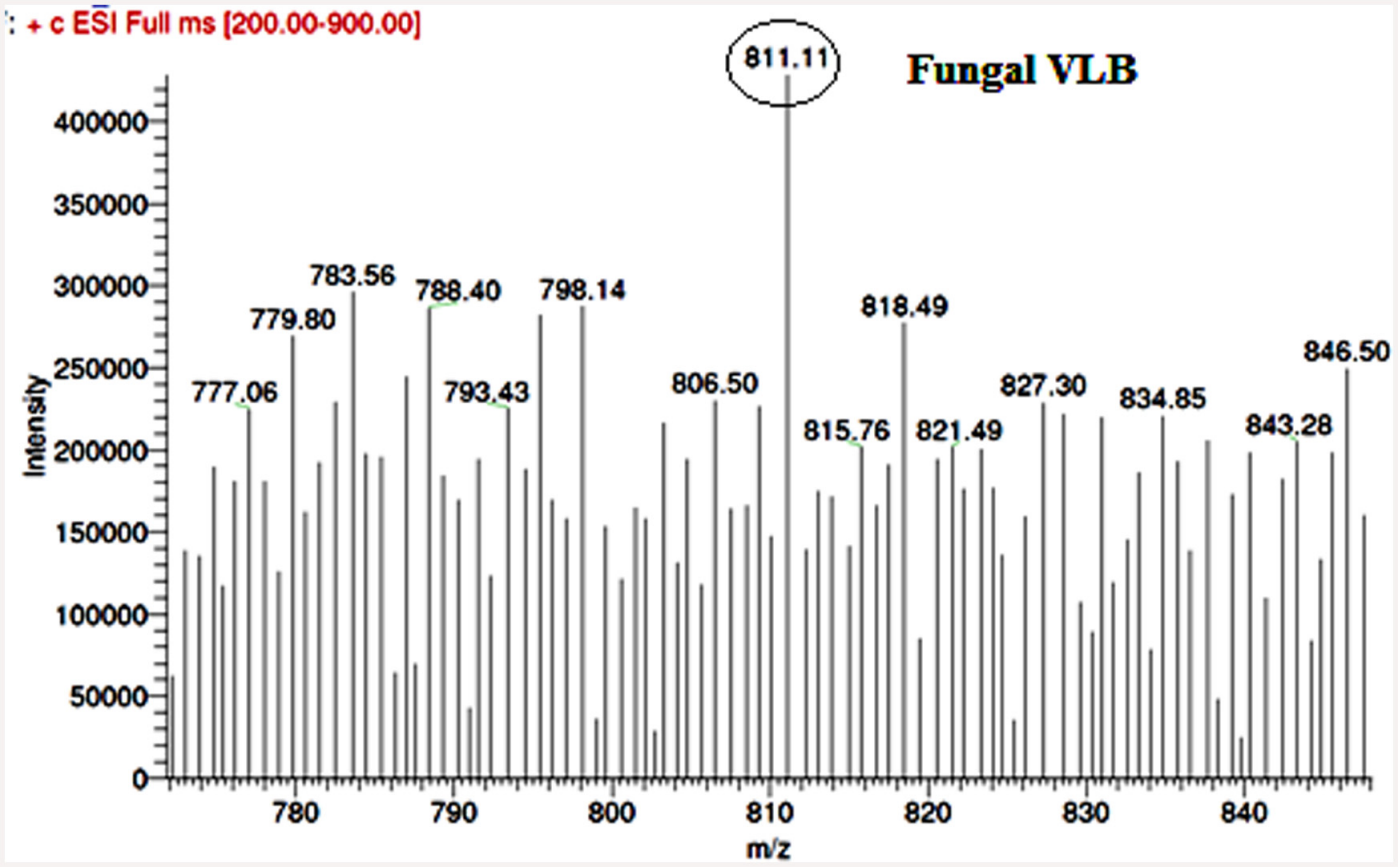
LC-ESI-MS analysis of fungal VBL. The mass spectrum of the fungal extract showed a (M+H^+^) peak at a molecular mass of 811.51, which was identical to that observed in the mass spectrum of the VBL standard.

Genomic PCR analysis suggested that *T*. *radicus*—CrP20 could produce TIAs. Chromatographic and spectroscopic data confirmed the presence of VCR and VBL in a 21-day-old culture of *T*. *radicus*—CrP20. TLC analysis of the crude fungal extract revealed the presence of VCR and VBL, which appeared as light brown spots when Dragendorff’s spray reagent was applied and co-migrated with the VCR (Rf 0.68) and VBL (Rf 0.75) standards (Fig 8). The LC-ESI-MS chromatogram of the crude extract, which showed the presence of molecular ions m/z 825.46 and 811.51 at the appropriate retention times, confirmed that the two compounds are VCR and VBL (Figs 9 and 10).

### Quantification of VCR and VBL production by *T*. *radicus*—CrP20 in different media

**Fig 11:**
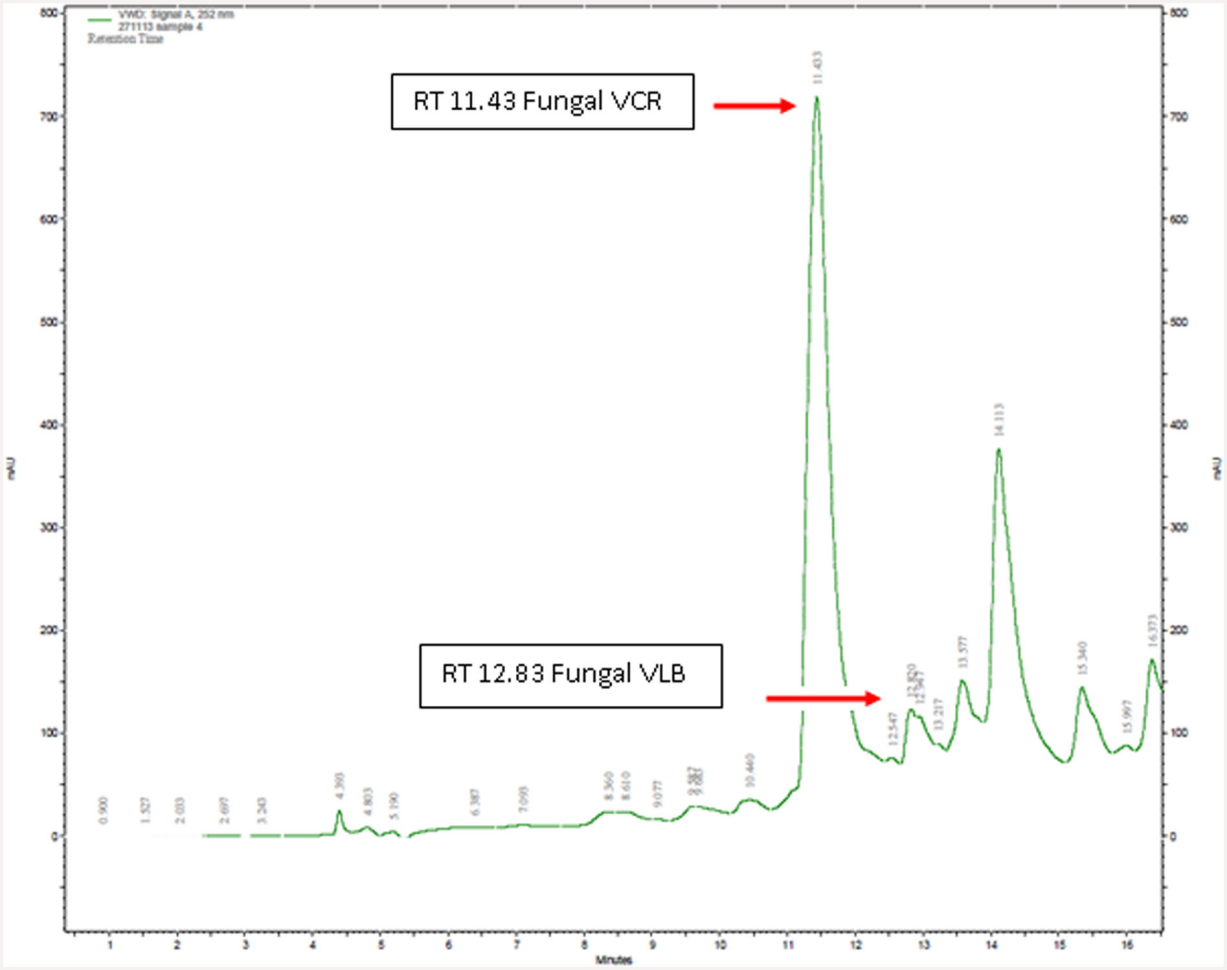
HPLC profile of *T*. *radicus*—CrP20 culture extract showing VCR (RT-11.43) and VBL (RT-12.83).

**Fig 12:**
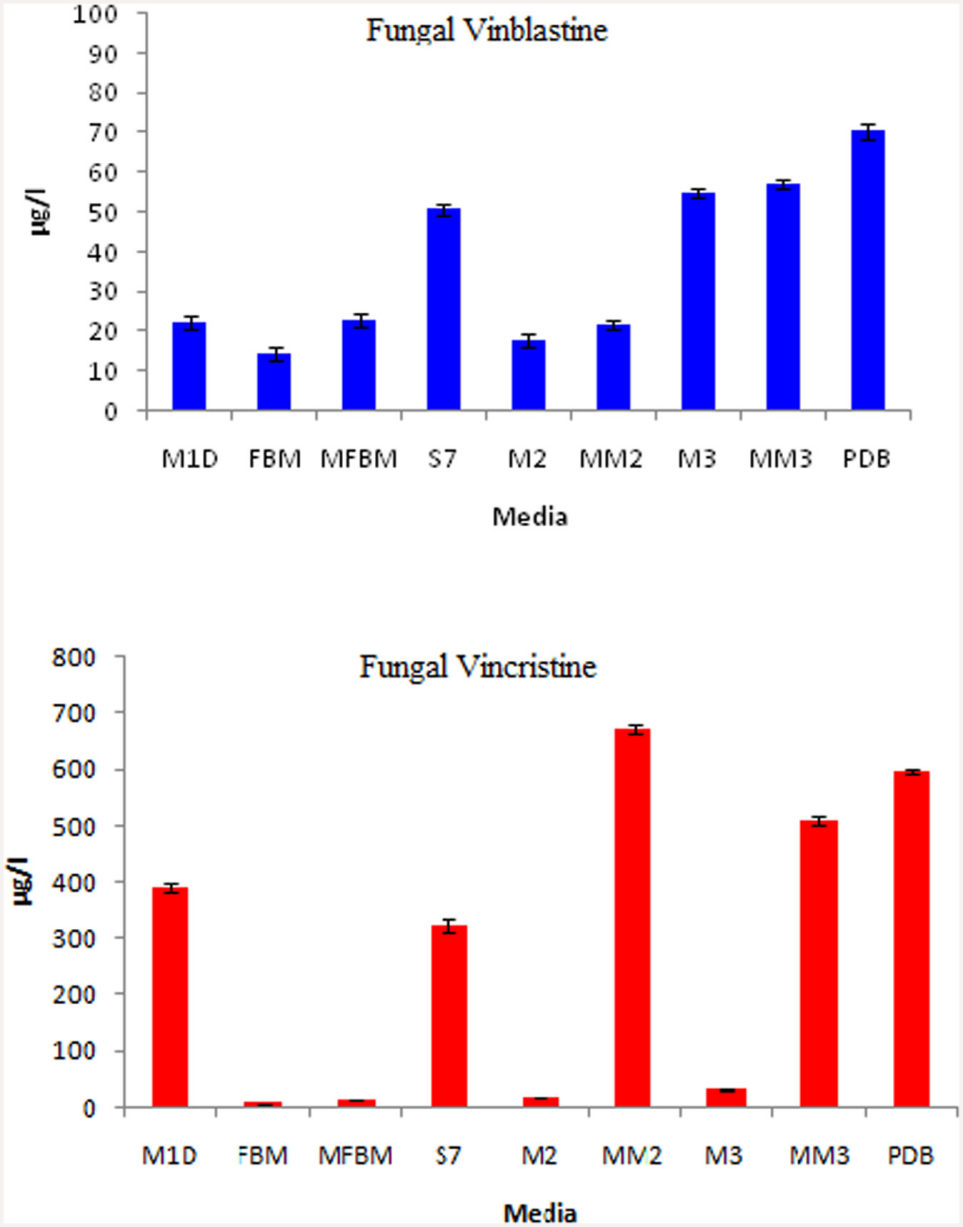
Production of VBL and VCR by *T*. *radicus*—CrP20 grown in different media.

The endophytic fungus *T*. *radicus*—CrP20 was grown in nine different growth media to determine the optimum conditions for producing VCR and VBL. After the indicated period of incubation, the cultures were extracted using the specific extraction protocol described in the methods section. The presence of VCR (RT—11.43 min) and VBL (RT—12.85 min) in the fungal extracts was confirmed by HPLC analysis based on the similarity of their retention times to the retention times of the standards. UV absorption analysis showed peaks at 252 nm for both compounds (Fig 11). The data obtained for the peak areas vs authentic VCR and VBL standard concentrations were used to construct standard curves and estimate fungal VCR and VBL yields. Yields of VCR from one liter of each medium were as follows: MM2–670 μg/l, PDB—596 μg/l, MM3–510.4 μg/l, M1D–390.4 μg/l, S7–323 μg/l, M3–30.8 μg/l, M2–17.49 μg/l, MFBM—13.7 μg/l and FBM—6.1 μg/l. Yields of VBL from one liter of each medium were as follows: PDB—70.5 μg/l, MM3–53.3 μg/l, M3–54.8 μg/l, S7–50.6 μg/l, MFBM—22.6 μg/l, M1D–22.3 μg/l, MM2–21.6 μg/l, M2–17.6 μg/l, and FBM—14.3 μg/l (Fig 12). The maximum concentration of VCR was obtained in MM2 medium (670 μg/l), and the lowest concentration was obtained in FBM (6.1 μg/l). The maximum concentration of VBL (70.5 μg/l) was found in PDB medium, and the lowest concentration was found in FBM (14.3 μg/l).

### Apoptotic activity of ‘fungal VCR’

#### Cytotoxic effects of ‘fungal VCR’ against human cancer cell lines

**Fig 13:**
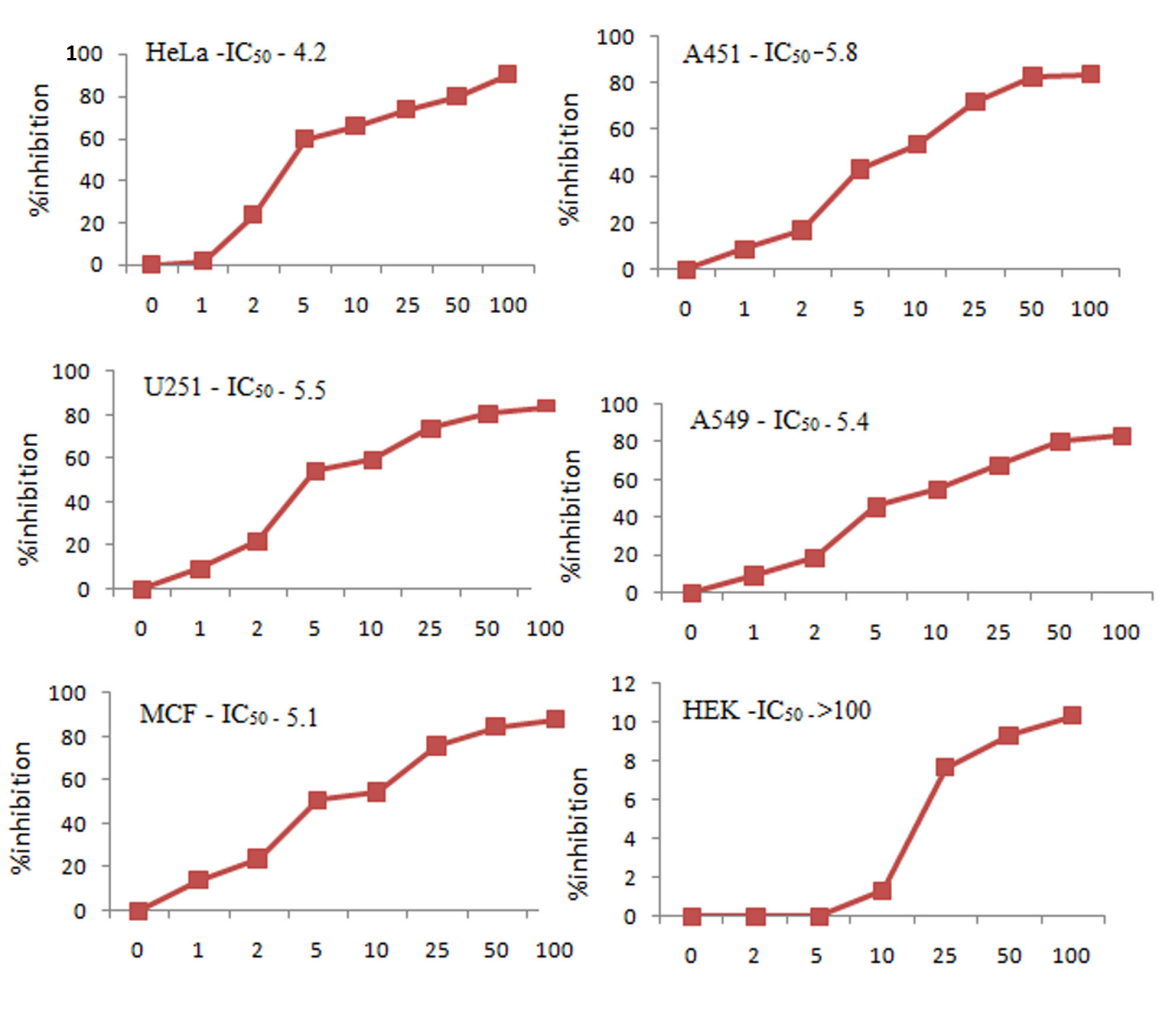
Cytotoxic activity of ‘fungal VCR’ in different cancer cell lines.

The cytotoxic effects of ‘fungal VCR’ against various cancer cell lines (i.e., HeLa, MCF7, U251, A549, and A431) and a normal cell line (HEK293) was tested by the MTT assay. Dose-dependent inhibition was observed in all cancer cell lines upon treatment with fungal VCR, with the following IC_50_ values: HeLa—4.2 μg/ml, MCF7–4.5 μg/ml, A549–5.5 μg/ml, U251–5.5 μg/ml and A431–5.8 μg/ml. In contrast, the normal cell line HEK293 was not inhibited (Fig 13). As HeLa cells appeared to be more susceptible, they were used in further studies of potential apoptotic activity.

#### Effect of ‘fungal VCR’ on cell cycle distribution in HeLa cells

**Fig 14:**
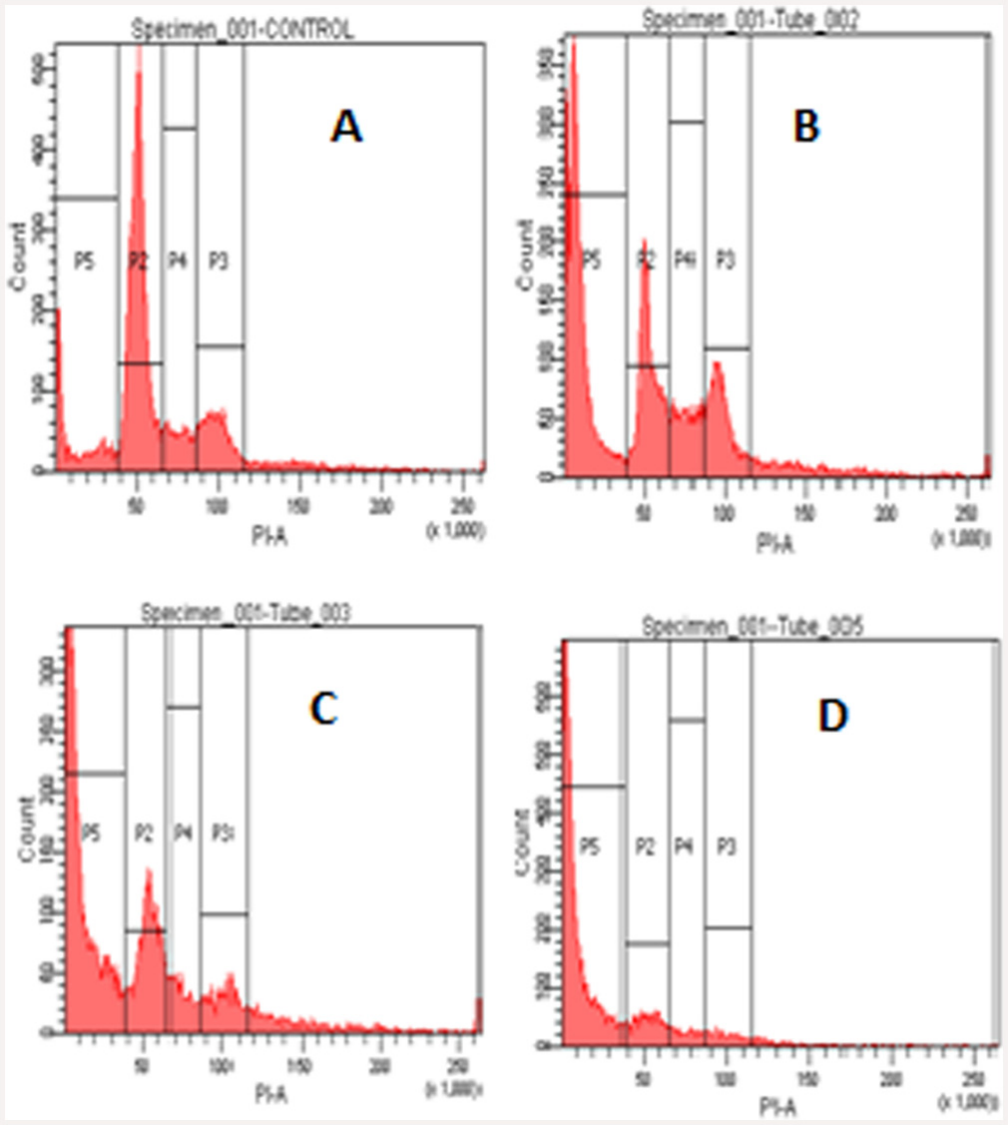
Cell cycle distribution of HeLa cells treated with different concentrations of ‘fungal VCR’. The sub-G0/G1, G1, S and G2/M phases are represented on the histogram as P5, P2, P4 and P3, respectively. A—control, B—fungal VCR (5 μg/ml), C—fungal VCR (10 μg/ml), D—fungal VCR (25 μg/ml) and E—percent apoptosis.

Flow cytometry was performed to determine the proportion of cells undergoing apoptosis or cell death when treated with different concentrations of ‘fungal VCR’. These studies illustrate the distribution of the cell population across various stages of the cell cycle (sub-G0/G1, G1, S and G2/M). Approximately 43%, 52% and 75% of the cells undergoing cell death were found to be in the sub G0/G1 stage following treatment with different concentrations of ‘fungal VCR’, and the percentage of apoptotic cells increased as the concentration of ‘fungal VCR’ increased (Fig 14). These results confirm that ‘fungal VCR’ induces cell death through the accumulation of cells in the sub-G0/G1 phase. Further experiments were conducted to establish whether the cell death was a result of apoptosis or necrosis.

#### Analysis of phosphatidyl serine externalization by HeLa cells treated with ‘fungal VCR’ using annexin V-FITC and PI dual staining

**Fig 15:**
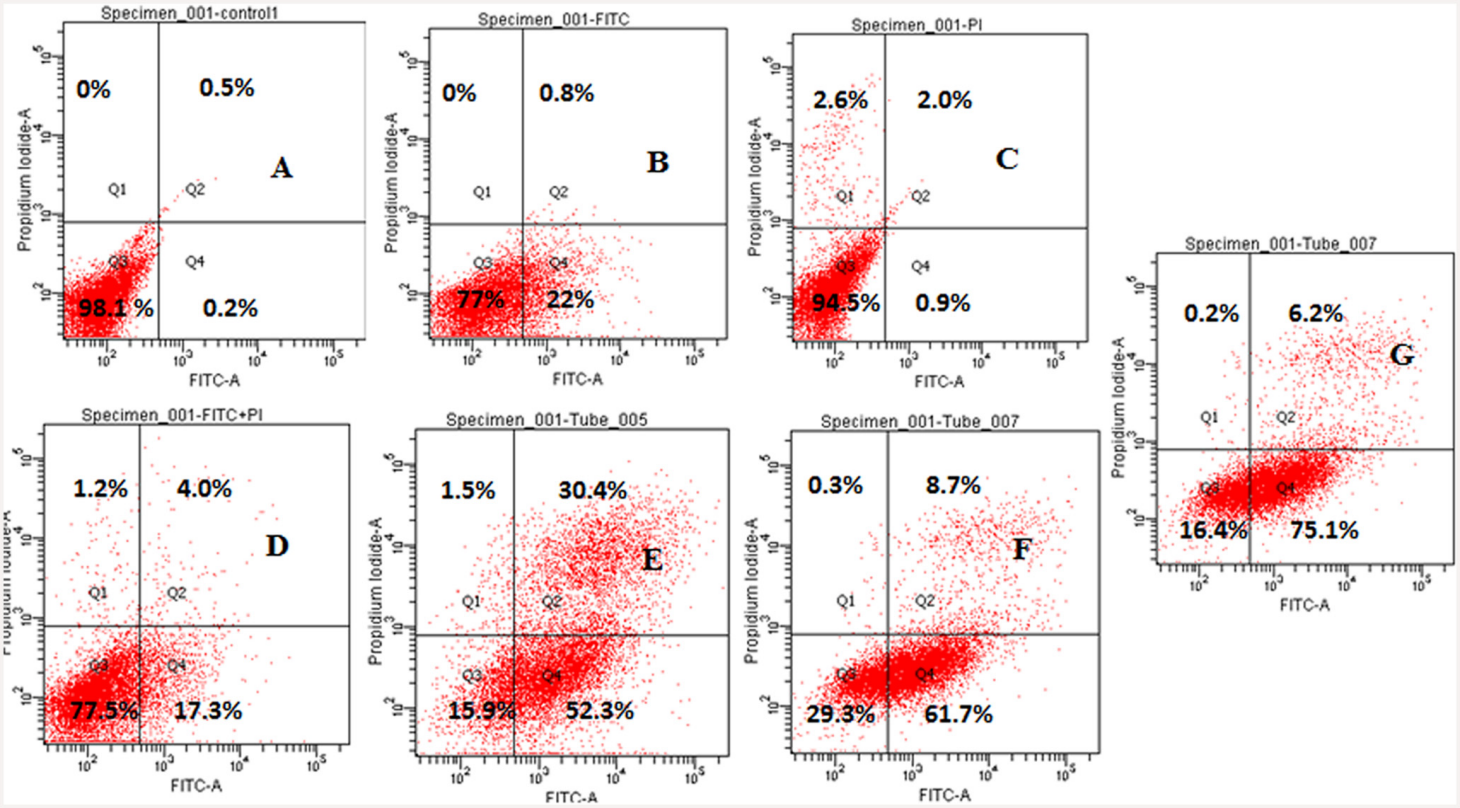
Induction of apoptosis in HeLa cells treated with different concentrations of ‘fungal VCR’, as determined by annexin V-FITC/PI dual staining. A- untreated cells, B—cells + FITC, C—cells + PI, D—cells + FITC + PI, E—cells + FITC + PI + fungal VCR (5 μg/ml), F—cells + FITC + PI + fungal VCR (10 μg/ml), G—cells + FITC + PI + fungal VCR (25 μg/ml) and H—percentage of cells undergoing early apoptosis.

The induction of apoptosis in HeLa cells by ‘fungal VCR’ was further confirmed by detecting externalized phosphatidyl serine at the plasma membrane using FITC-conjugated annexin V. Because annexin V also binds to necrotic cells, the cells were counterstained with PI to distinguish between apoptotic and necrotic cells. HeLa cells were treated with different concentrations of fungal VCR for 2 h and stained with annexin V-FITC and propidium iodide. FACScan analysis of the untreated cells revealed that the cells were negative for annexin V-FITC and PI, indicating that they were viable and not undergoing apoptosis. Upon treatment with different concentrations (5, 10 and 25 μg/ml) of fungal VCR, the cells began to distribute into four different populations. The following are the quadrant representatives: Q1—dead cells (PI positive); Q2—cells undergoing late apoptosis or necrosis (annexin V-FITC and PI positive); Q3—healthy cells (annexin V-FITC and PI negative) and Q4—cells undergoing early apoptosis (annexin V-FITC positive and PI negative). As the concentration of ‘fungal VCR’ increased, the accumulation of cells in Q2 decreased, which is suggestive of late apoptosis or necrosis. In Q4, the accumulation of cells increased, indicating that the HeLa cells were undergoing early apoptosis, which steadily increased as the concentration of ‘fungal VCR’ increased (52.3, 61.7 and 75%). A remarkable observation was that ‘fungal VCR’ at a low concentration of 5 μg/ml elicited 52.3% early apoptosis in HeLa cells (Fig 15). The results strongly support the ability of ‘fungal VCR’ to induce apoptosis in HeLa cells.

#### Induction of mitochondrial membrane depolarization in HeLa cells by ‘fungal VCR’

**Fig 16:**
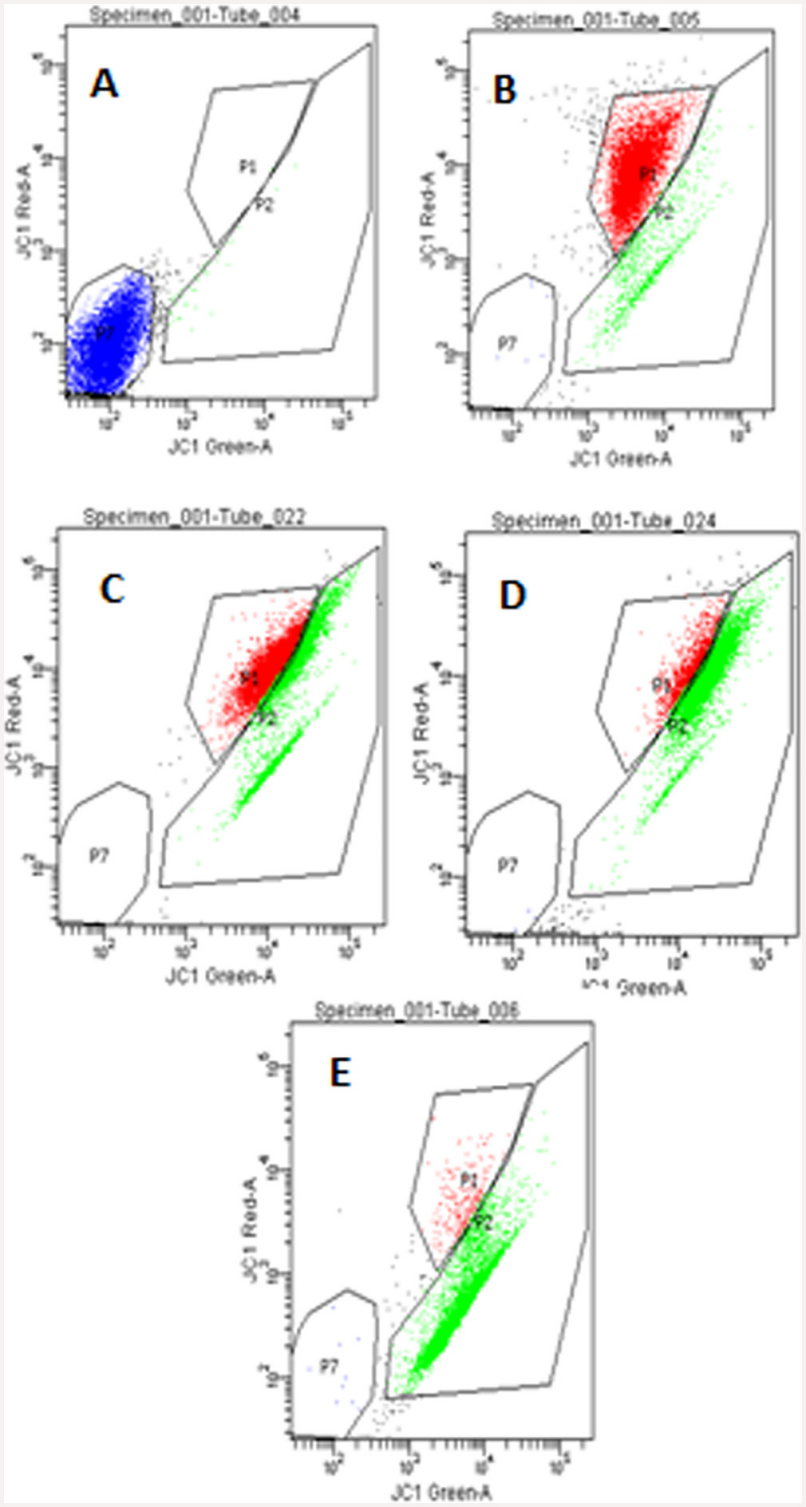
Induction of mitochondrial membrane depolarization in HeLa cells treated with various concentrations of ‘fungal VCR’. A—cells alone, B—cells + JC1 stain, C—cells + JC1 + 25 μM valinomycin (+ ve control), D—cells + JC1 + fungal VCR (5 μg/ml), E—cells + JC1 + fungal VCR (10 μg/ml), F—cells + JC1 + fungal VCR (25 μg/ml), G—standard VCR (2 nM) and H—percent loss of mitochondrial membrane depolarization.

To understand the effect of different concentrations of ‘fungal VCR’ on the mitochondrial apoptotic pathway, we measured the loss in mitochondrial membrane potential in HeLa cells via JC-1 staining. HeLa cells treated with fungal VCR showed a dose-dependent decrease in mitochondrial membrane potential (P2 region; Fig 16), whereas control cells did not exhibit a significant loss in potential (P7 region). In addition, considerable mitochondrial membrane depolarization was observed in the positive control and standard VCR-treated cells. Mitochondrial membrane depolarization is an early apoptotic event followed by the release of cytochrome *c* and the activation of caspase 9, eventually leading to apoptotic cell death due to the action of downstream caspases. These results suggest that ‘fungal VCR’ induces apoptosis, as some chemotherapeutic agents do, through the mitochondrial pathway in HeLa cells.

#### DNA fragmentation in HeLa cells due to ‘fungal VCR’

**Fig 17:**
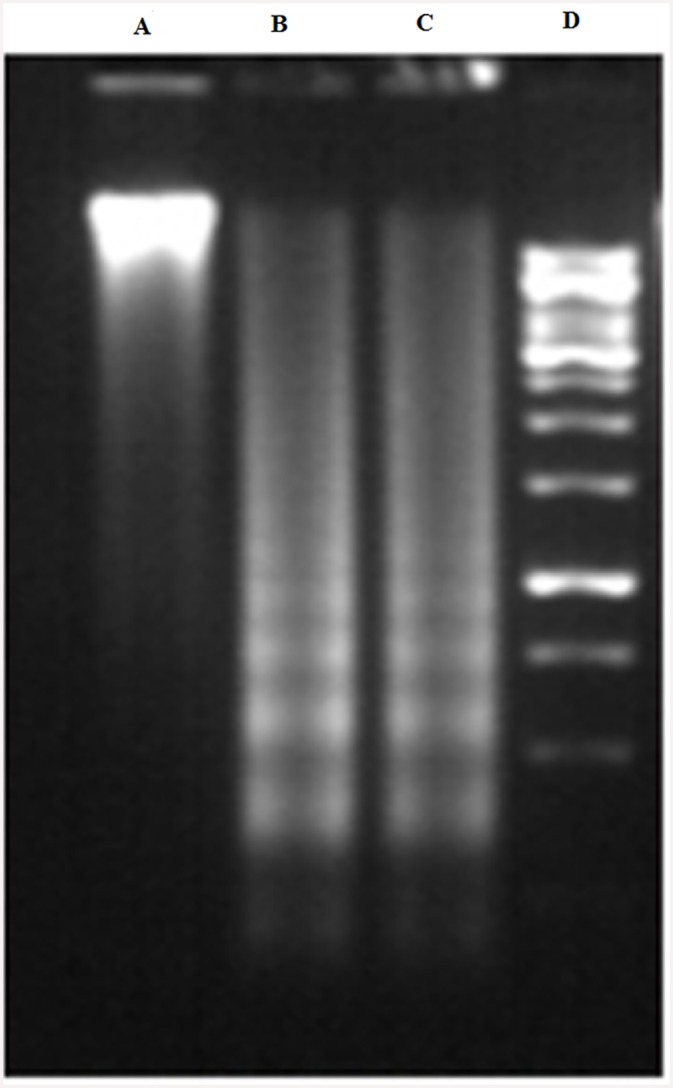
DNA fragmentation in HeLa cells treated with ‘fungal VCR’. A—control, B—cells treated with fungal VCR (10 μg/ml), C—cells treated with fungal VCR (25 μg/ml) and D—1-kb DNA ladder.

DNA fragmentation is an important feature in cells undergoing apoptosis. To confirm that ‘fungal VCR’ induces cell death in HeLa cells via apoptosis and not necrosis, DNA fragmentation patterns in treated cells were analyzed. The activation of endogenous endonucleases results in the cleavage of chromatin into inter-nucleosomal fragments of approximately 180–200 bp; it is believed that DNA fragmentation is carried out by caspase-activated DNase (CAD), leading to the cleavage of nuclear DNA into multiple fragments with low molecular weights. HeLa cells treated with two different concentrations of ‘fungal VCR’ for approximately 2 h showed inter-nucleosomal DNA fragmentation in the form of a DNA ladder, typical of apoptotic DNA fragmentation (Fig 17). The precise DNA fragmentation and the absence of a DNA smear due to non-specific DNA degradation indicate the apoptosis-inducing ability of ‘fungal VCR’.

Similar apoptotic experiments with TLC-purified fungal VBL have not been successful to date because researchers have been unable to obtain adequate amounts of VBL from fungi.

## Discussion

Endophytes are mutualistic symbionts of healthy plants and are known to produce a wide range of bioactive compounds with a broad spectrum of activities. In the present study, 22 endophytic fungi were isolated from various parts of *C*. *roseus* plants and grouped into 10 genera based on partial ITS sequencing. Among the *A*. *niger* isolates, two were obtained from the petiole, and the others were obtained from the stem. Of the three *T*. *radicus* isolates obtained, two were derived from the leaf, and one was derived from the stem. The remaining genera were procured from stem tissue only. The flower and stem parts of *C*. *roseus* harbored most of the fungi. The results of the present study indicate that *Alternaria* and *Colletotrichum* species predominate in the petiole and petal regions of the flower. In a previous study, thirteen fungal genera were isolated from *C*. *roseus* leaf, stem and root tissues, with *Alternaria*, *Cladosporium and Aspergillus* colonizing roots and *Curvularia*, *Biopolaris*, *Alternaria* and *Aspergillus* colonizing leaves [34]. In contrast, our study showed that *Alternaria* was more prevalent in all the tissues of *C*. *roseus*; the presence of the same genus in the leaves and nodal regions, but not the flower parts, of this plant was previously reported [35]. In general, universal primers for ITS regions are employed to identify fungal genera and species because they target conserved sequences. In the present study, ITS1 and ITS4 primers [36] were used for amplification of the ITS-5.8S-ITS2 region. The amplicons were later sequenced and compared with reported sequences in GenBank to taxonomically identify the fungal isolates.

Because the aim of the present investigation was to search for an alternative and viable source of VCR and VBL, all 22 of the endophytic fungi isolated were screened for the TDC gene using a molecular approach. TDC is the key enzyme in the early steps of the TIA biosynthetic pathway. Tryptamine, the product of TDC, condenses with secologanin to yield strictosidine, the precursor of the pharmaceutically important alkaloids VCR and VBL [37]. TIAs consist of an indole moiety (provided by tryptamine) and a terpenoid component (derived from the iridoid glucoside secologanin). TDC is encoded by a single gene in *C*. *roseus*, and *T*. *radicus*—CrP20 was the only isolate that showed amplification of this gene. It is believed that the horizontal transfer of some biosynthetic genes from the plant to the fungus occurs during their symbiotic association, resulting in the ability of the fungus to produce host plant-specific compounds. Indeed, plant biosynthetic genes for taxol and camptothecin are known to be present in endophytes that were identified as candidates for the production of these compounds [38, 39]. This study confirmed the presence of the TDC gene in *T*. *radicus*—CrP20 and the presence of VCR and VBL in the fungal extract.

Microorganisms produce secondary metabolites in response to environmental stress or nutrient availability, and manipulation of synthetic media and fermentation protocols can also tremendously affect the production and diversity of secondary metabolites. The addition or depletion of one or more nutrients may result in substantial differences in secondary metabolite production by microorganisms [40]. For example, the yield of penicillin was shown to increase when the culture conditions of *Penicillium* species were improved [41]. Therefore, selective changes in culture conditions can be explored with the aim of optimizing biosynthetic pathways [42]. In the present study of *T*. *radicus*—CrP20, a marked increase in VCR was observed in modified M2 and M3 media (MM2 and MM3).

Cancer chemotherapy drugs usually exert cytotoxic effects on malignant cells by inducing apoptosis [43]. Major apoptotic events include cell cycle arrest and the formation of reactive oxygen species, leading to loss of mitochondrial membrane potential and DNA fragmentation. It is believed that caspase 3, a major downstream effector of apoptosis, mediates proteolytic cleavage of poly (ADP ribose) polymerase (PARP), leading to DNA fragmentation. These apoptotic events, accompanied by the potent growth inhibition of HeLa cells, were detected when the cells were treated with partially purified VCR from *T*. *radicus*—CrP20. A decrease in mitochondrial transmembrane potential in HeLa cells was also observed after treatment with ‘fungal VCR’, similar to what was observed with standard VCR, vinca alkaloids [44] and other anti-cancer drugs.

## Conclusions

We report for the first time that of the 22 endophytic fungi isolated from *C*. *roseus*, the endophytic fungus *T*. *radicus*—CrP20 exhibits constitutive expression of the TDC gene and produces VCR and VBL without the addition of external precursors, with optimal yields in MM2 and PDB media, respectively. Fungal VCR and VBL in extracts were characterized by TLC and LC-ESI-MS, and ‘fungal VCR’ was able to induce apoptotic cell death in HeLa cells. This fungus therefore has the biotechnological potential to produce large quantities of VCR and VBL.
