# Correction: An Endophytic Fungus, *Talaromyces radicus*, Isolated from *Catharanthus roseus*, Produces Vincristine and Vinblastine, Which Induce Apoptotic Cell Death

**DOI:** 10.1371/journal.pone.0153111

**Published:** 2016-04-05

**Authors:** Padmini P. C. Palem, Gini C. Kuriakose, Chelliah Jayabaskaran

Figs 9, 10, 14, 15 and 16 are incorrect. Although the legends are accurate, the final formatted version are not present. The authors have provided the corrected figures here.

**Fig 9:**
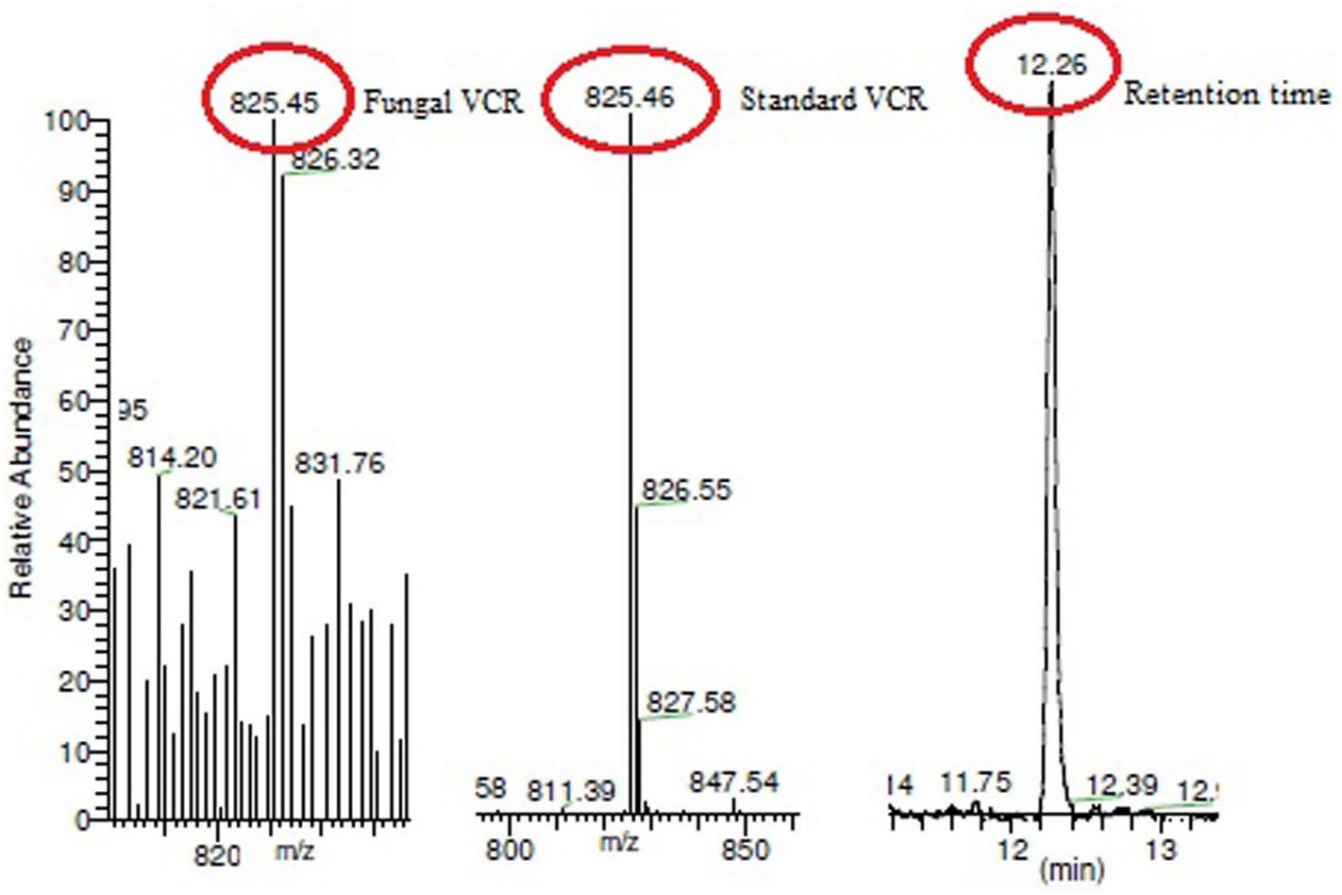
LC-ESI-MS analysis of fungal VCR. The mass spectrum of the fungal extract showed a (M+H+) peak at a molecular mass of 825.46, which was identical to that observed in the mass spectrum of the VCR standard.

**Fig 10:**
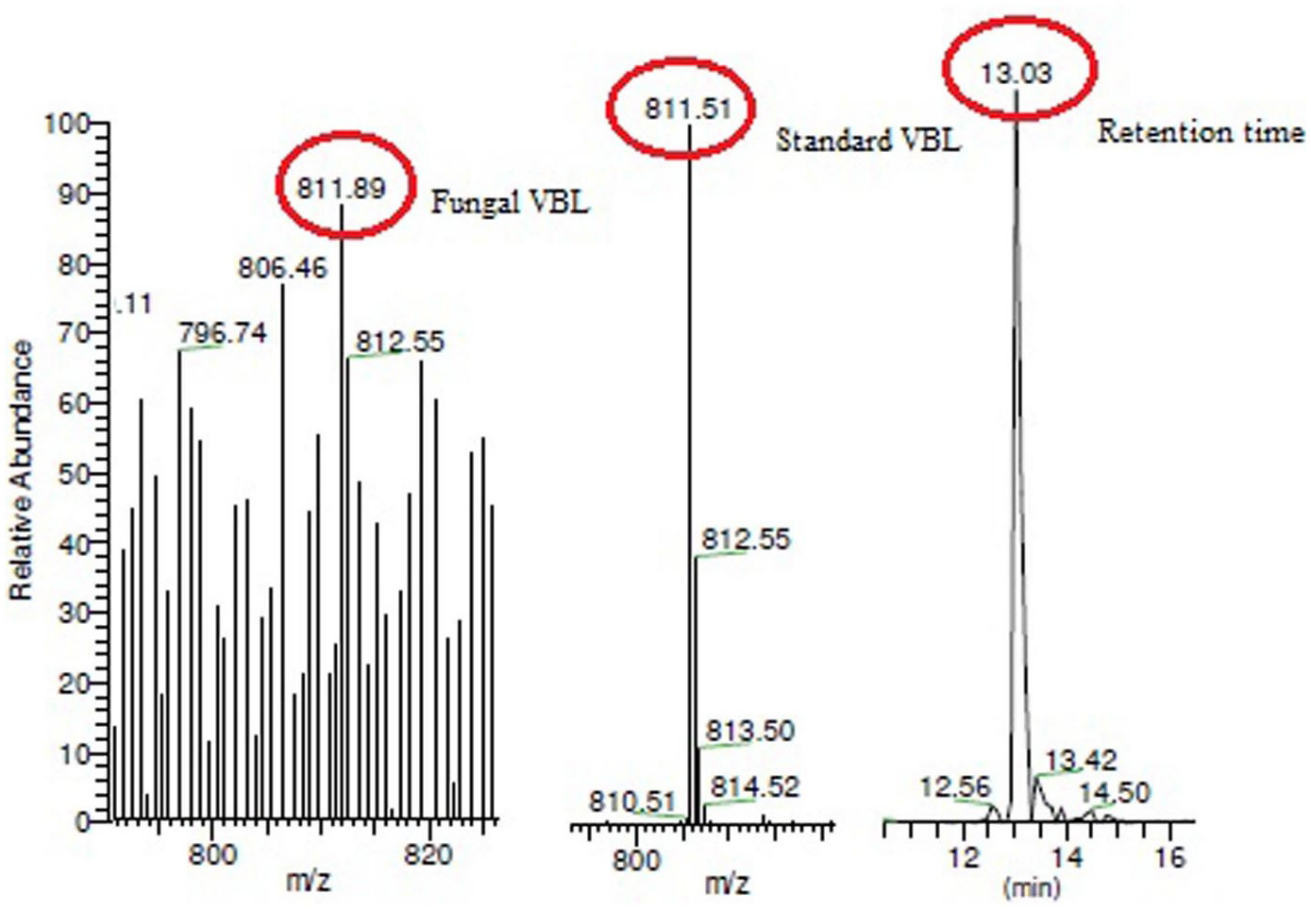
LC-ESI-MS analysis of fungal VBL. The mass spectrum of the fungal extract showed a (M+H+) peak at a molecular mass of 811.51, which was identical to that observed in the mass spectrum of the VBL standard.

**Fig 14:**
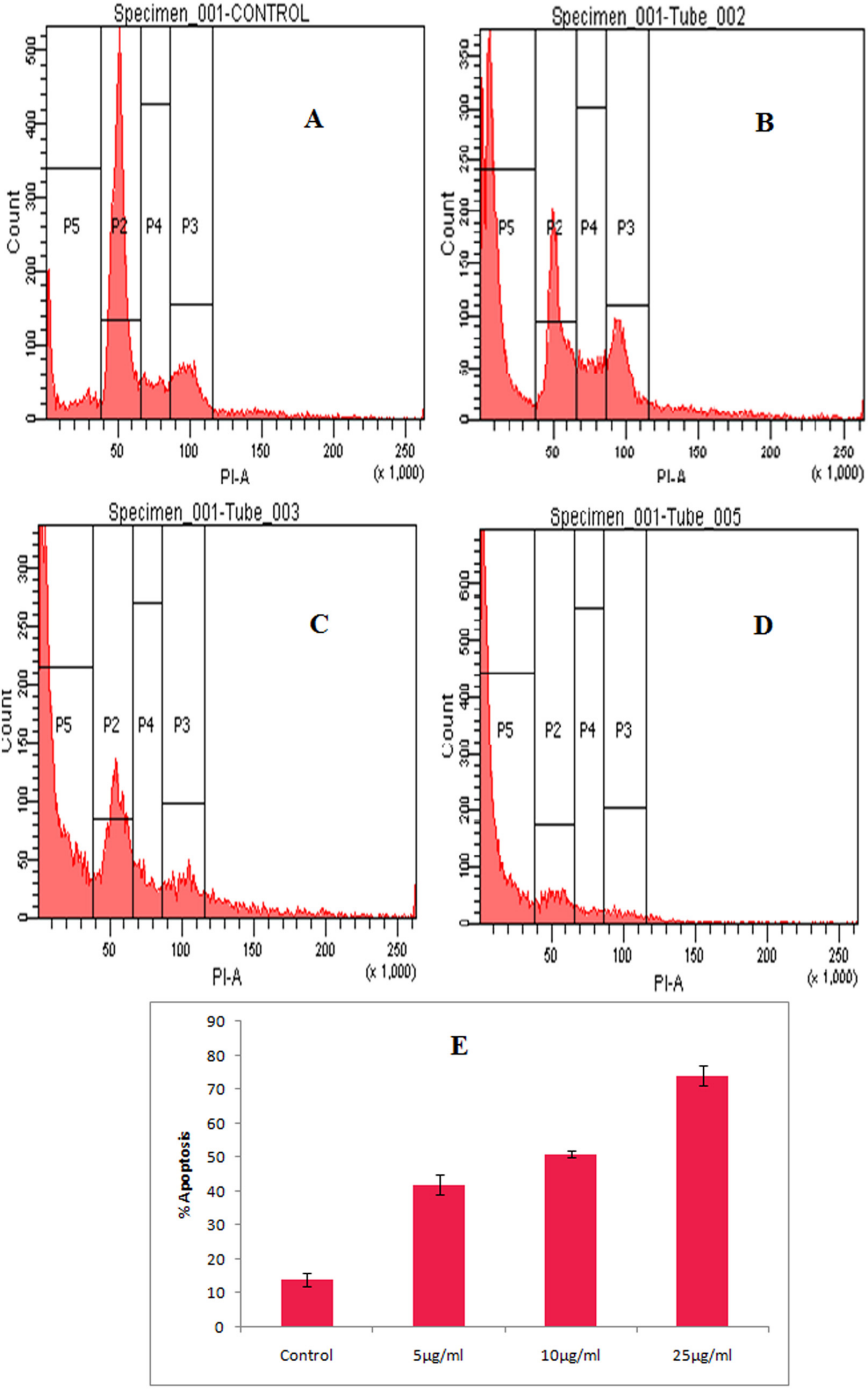
Cell cycle distribution of HeLa cells treated with different concentrations of ‘fungal VCR’. The sub-G0/G1, G1, S and G2/M phases are represented on the histogram as P5, P2, P4 and P3, respectively. A—control, B—fungal VCR (5 μg/ml), C—fungal VCR (10 μg/ml), D—fungal VCR (25 μg/ml) and E—percent apoptosis.

**Fig 15:**
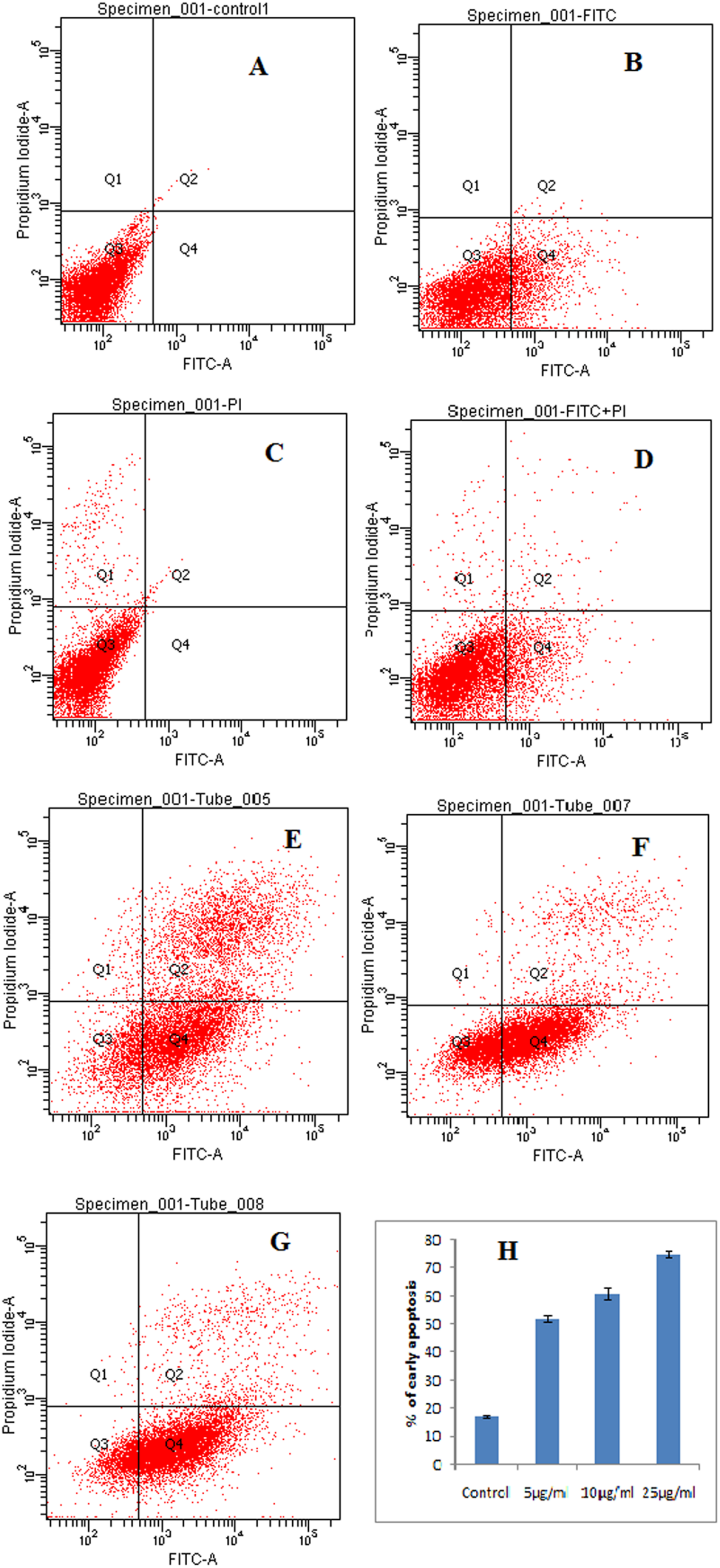
Induction of apoptosis in HeLa cells treated with different concentrations of ‘fungal VCR’, as determined by annexin V-FITC/PI dual staining. A—untreated cells, B—cells + FITC, C—cells + PI, D—cells + FITC + PI, E—cells + FITC + PI + fungal VCR (5 μg/ml), F—cells + FITC + PI + fungal VCR (10 μg/ml), G—cells + FITC + PI +

**Fig 16:**
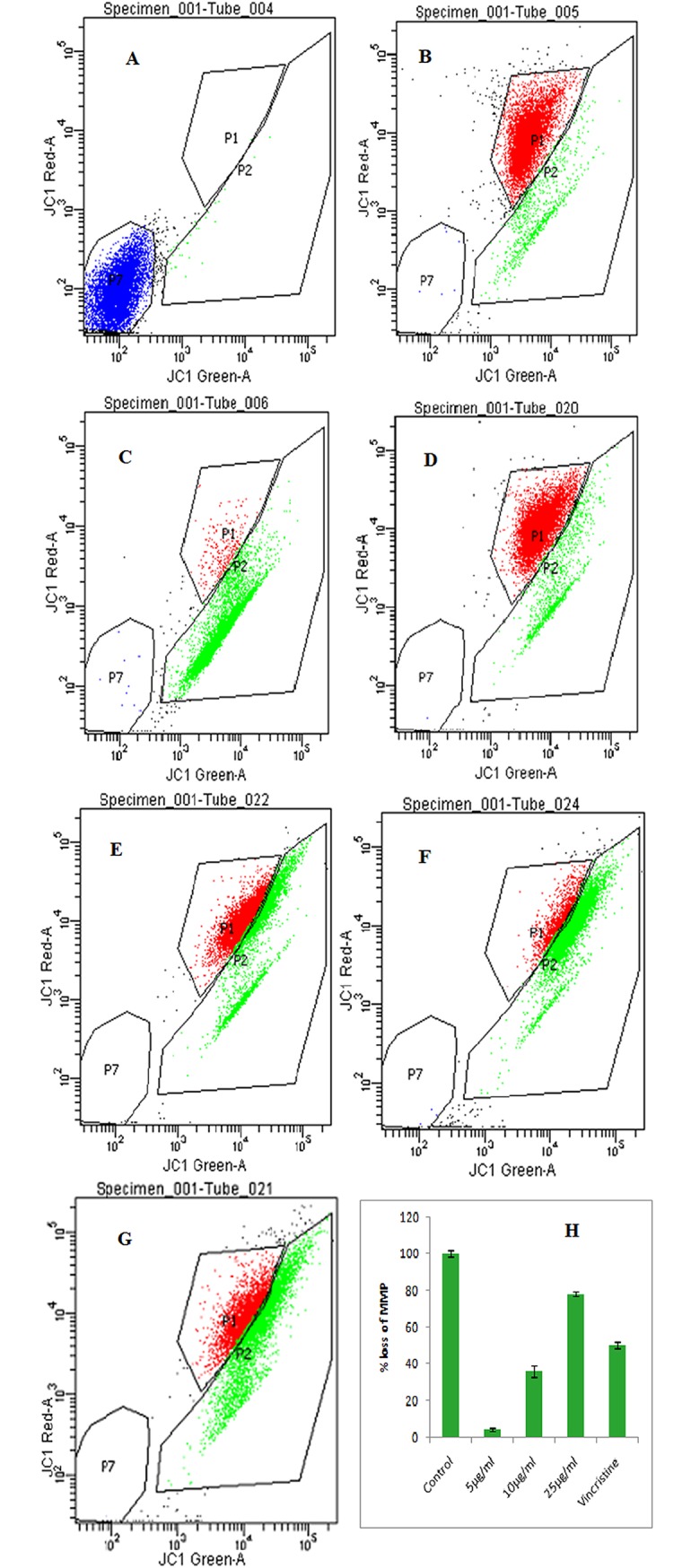
Induction of mitochondrial membrane depolarization in HeLa cells treated with various concentrations of ‘fungal VCR’. A—cells alone, B—cells + JC1 stain, C—cells + JC1 + 25 μM valinomycin (+ ve control), D—cells + JC1 + fungal VCR (5 μg/ml), E—cells + JC1 + fungal VCR (10 μg/ml), F—cells + JC1 + fungal VCR (25 μg/ml), G—standard VCR (2 nM) and H—percent loss of mitochondrial membrane depolarization.
